# The Impact of the COVID-19 Pandemic and Societal Infection Control Measures on Children and Adolescents' Mental Health: A Scoping Review

**DOI:** 10.3389/fpsyt.2021.711791

**Published:** 2021-09-06

**Authors:** Jamile Marchi, Nina Johansson, Anna Sarkadi, Georgina Warner

**Affiliations:** Child Health and Parenting (CHAP), Department of Public Health and Caring Science, Uppsala University, Uppsala, Sweden

**Keywords:** COVID-19, pandemic, children, adolescents, mental health, scoping review

## Abstract

**Background:** The COVID-19 pandemic is primarily a crisis that affects people's physical health. However, it is well-known from previous epidemics and pandemics that there are other indirect negative impacts on mental health, among others. The purpose of this scoping review was to explore and summarise primary empirical research evidence on how the COVID-19 pandemic and societal infection control measures have impacted children and adolescents' mental health.

**Methods:** A literature search was conducted in five scientific databases: PubMed, APA PsycINFO, Web of Science, CINHAL, and Social Science Premium Collection. The search string was designed using the Population (0–18 years), Exposure (COVID-19), Outcomes (mental health) framework. Mental health was defined broadly, covering mental well-being to mental disorders and psychiatric conditions.

**Results:** Fifty-nine studies were included in the scoping review. Of these, 44 were cross-sectional and 15 were longitudinal studies. Most studies reported negative impact of the COVID-19 pandemic on child and adolescent mental health outcomes, yet the evidence was mixed. This was also the case for studies investigating societal control measures. Strong resilience, positive emotion regulation, physical activity, parental self-efficacy, family functioning and emotional regulation, and social support were reported as protective factors. On the contrary, emotional reactivity and experiential avoidance, exposure to excessive information, COVID-19 school concerns, presence of COVID-19 cases in the community, parental mental health problems, and high internet, social media and video game use were all identified as potentially harmful factors.

**Conclusions:** Due to the methodological heterogeneity of the studies and geographical variation, it is challenging to draw definitive conclusions about the real impact of the COVID-19 pandemic on the mental health of children and adolescents. However, the existing body of research gives some insight to how parents, clinicians and policy makers can take action to mitigate the effects of COVID-19 and control measures. Interventions to promote physical activity and reduce screen time among children and adolescents are recommended, as well as parenting support programs.

## Introduction

The outbreak of a new respiratory syndrome, declared as coronavirus disease 2019 (COVID-19), was first identified in Wuhan, China, in December 2019 (1) and continued to spread rapidly around the world. On March 11th 2020, the World Health Organisation (WHO) declared COVID-19 as a global pandemic that had spread to 114 countries. COVID-19 showed a high transmission ability compared with SARS (severe acute respiratory syndrome) and MERS (Middle East respiratory syndrome); however, on the other hand, COVID-19 showed a lower mortality rate (4.5–6.0%) when compared to SARS (9.6%), and MERS (34.4%) (1).

It is believed the virus that causes COVID-19 spreads mainly from person to person. As an attempt to slow down the spread of the virus, in mid-March 2020, many countries took preventive measures such as social distancing and quarantine. By September–October 2020, after a relaxation of lockdowns and the population's precautionary behaviours, indicators of a second wave were emerging in many European countries. From December 2020, international vaccination roll-out programmes commenced. Now more recently, in February–March 2021, there are concerns regarding a third wave. Restrictions are coming back but, in some countries, not as strict as in the first wave. By end of March 2021, more than 2.8 million people in the world had died from the virus and nearly 130 million had reported infection.

The COVID-19 pandemic is primarily a crisis that affects people's physical health, but it is well-known from previous epidemics and pandemics that the event, including societal measures to control infection, also affects the mental health of the population directly and indirectly (2), among a number of other potential negative outcomes. The societal infection control measures have proved to be successful controlling the spread of the virus (3); however, at the same time, the interruption of the daily routine of children, adolescents and their families has impacted their lives (4).

COVID-19 is an unprecedented global crisis compared to the most recent epidemics and children and adolescents are experiencing a prolonged state of physical isolation from their peers, teachers, extended families, and community networks (5). Added to children and adolescents' fear of personal and family member infection, there are other pandemic-related factors that could affect mental health outcomes such as family job or financial loss and social isolation due to infection containment measures (5).

It is crucial to understand and investigate the real impact of the COVID-19 pandemic and related societal measures to develop, adapt, and implement mitigation strategies for these outcomes in order to help children and adolescents' mental health and well-being during these stressful times as well as for future similar pandemics.

The aim of this scoping review was to explore and summarise primary research evidence on how the COVID-19 pandemic and societal infection control measures (such as “lockdown,” quarantine, social isolation, social distancing, and school closures) aimed at minimising the spread of the disease have impacted children and adolescents' mental health, from birth up to 18 years.

## Methods

The scoping review followed the methodological framework as described by Arksey and O'Malley (6). The framework sets out five steps: (i) identifying the research question(s); (ii) identifying relevant studies; (iii) study selection; (iv) charting the data; and (v) collating, summarising and reporting the results. Yet, Arksey and O'Malley state that researchers should engage with each stage in a reflexive way (6). In other words, the process should not be considered linear but iterative. This means steps can be revisited, if needed.

### Identifying the Research Questions

In order to thematically construct the account of existing literature, specific research questions were developed through discussion among the authors:

1. Has the COVID-19 pandemic and societal infection control measures impacted child and adolescent mental health?
2. What is the evidence from different geographical regions?
3. Are there any protective factors associated with a lower likelihood of mental health problem outcomes?
4. Are there any factors associated with a higher likelihood of mental health problem outcomes?

### Identifying Relevant Studies

The literature search was conducted in five scientific databases: PubMed, APA PsycINFO, Web of Science, CINHAL, and Social Science Premium Collection by the University librarian. The search was performed on 4th December 2020. The search string was designed using the Population, Exposure, Outcomes (PEO) framework and adapted for each database by the University librarian (Appendix 1). Study identification was conducted in an iterative way (6), with a second search performed on 5th May 2021 using the same search string and databases as in the first search. The aim was to update the scoping review. As a prioritisation strategy, the second search focused only on longitudinal studies.

### Study Selection

Only studies written in English were included. Further eligibility criteria were developed based on the aims of the review, which are summarised in Table 1. Reference management software was used to import and collate studies from the different databases. All non-duplicate references were screened in a staged process: if the inclusion/exclusion criteria were unclear from the title, the abstract was reviewed. Similarly, if the abstract did not provide sufficient detail then the full text was reviewed.

**Table 1:** Scoping review eligibility criteria.

	**Include**	**Exclude**
Population	Children and adolescents (mean age <18 years)	Adults (mean age >18 years), including college and university students Aggregated data, for which it is not possible to extract child/adolescent data, or the N for the age range is very small Clinical samples e.g., physical or mental health conditions and/or disabilities
Exposure	COVID-19 pandemic, including related societal control measures	
Comparison	No restrictions	
Outcomes	Mental health. Defined broadly, ranging from mental well-being to emotional disturbance and symptoms of clinical disorder	Illness and hospital admissions indirectly related to COVID-19 infection
Study design	Empirical study with primary data and quantitative analysis	Qualitative studies Books, book chapters, editorials, opinion pieces, indices, research letters, conference reports and posters, case-reports, case-series, pre-print, letter to the editor, commentary, and pilot studies Systematic reviews, scoping reviews, narrative reviews rapid reviews, and other reviews

### Charting the Data

Data from the selected studies were extracted into a data charting form using the database programme Excel. This included: author(s); year of publication; study location; aims; methodology; outcome measures; and important results.

### Collating, Summarising, and Reporting the Results

As scoping reviews seek to present an overview of all material reviewed (6), tables were constructed of *all* cross-sectional studies (Table 2) and *all* longitudinal studies (Table 3) included in the review. The reason for highlighting the methodological difference between studies was 2-fold. First, it is a logical and informative way to organise the studies when reporting the overall field of research. Second, it was used as a prioritisation strategy for the review update, as longitudinal studies are more likely to indicate causality, which is important when issuing recommendations based on the data. Within each table, the studies were organised by continent and country.

**Table 2:** Overview of cross-sectional studies organised by continent and country.

	**Author, year, DOI**	**Country, continent**	**Primary aim/main objective**	**Exposure**	**Mental health variables—child and adolescent**	**Child and adolescent outcomes relevant to the review**
**Asia**	**Yeasmin et al**. **(****7****)**. https://doi.org/10.1016/j.childyouth.2020.105277	Bangladesh, Asia	“To investigate the impact of the COVID-19 pandemic on mental health and determining the associated factors among children of Bangladesh.”	COVID-19 pandemic	Mental health	Children were classified into four groups: 43% of children had subthreshold mental health disturbances, 31% had mild disturbances, 19% moderate disturbances, and 7% severe disturbances. Child mental health was affected by parental mental health, as well as parents' attitudes toward the child.
	**Chen et al**. **(****8****)**. https://doi.org/10.1186/s12992-020-00627-7	China, Asia	“To understand whether there is a clinically significant difference in anxiety, depression, and parental rearing style when comparing adolescents from Wuhan and other cities in China.”	COVID-19 pandemic	Depression and anxiety	Wuhan adolescents' anxiety symptoms were significantly higher than in other urban areas, but not their depressive symptoms. Wuhan adolescents' parents might be under higher stress than other urban areas, and that, in turn, would have a negative effect on the outcome of some adolescents' emotional state.
	**Dong et al**. **(****9****)**. https://doi.org/10.3389/fpsyt.2020.00751	China, Asia	“To assess Internet use characteristics and objectively examine the potential psychological factors associated with Internet addiction during the COVID-19 epidemic.”	Internet/technology use	Depression, anxiety, and stress	The prevalence of depression, anxiety, and stress were found to be 18, 16, and 7%, respectively. Depression and stress were significantly correlated with internet addiction scores.
	**Duan et al**. **(****10****)**. https://doi.org/10.1016/j.jad.2020.06.029	China, Asia	“To assess the current status of mental health issues among children and adolescents and to analyse its influencing factors to provide scientific guidance to psychological professionals and the government in formulating targeted policies.”	COVID-19 pandemic	Depression, anxiety, and coping	55 and 36% of participants reported the pandemic has affected their learning and graduation, respectively. Among all respondents, 22% had scores above the threshold for clinical depressive symptoms, and 6% for Internet addiction.
	**Kang et al**. **(****11****)**. https://doi.org/10.2991/jegh.k.200908.001	China, Asia	“To investigate whether physical activity and sedentary behaviour of adolescents were related to their mood states and mental health during the isolation period caused by COVID-19 pandemic.”	Physical activity and sedentary behaviour	Mood state	Girls and students in Grade 3 Senior High School had higher level of mood disturbance. More physical activity was associated with improved mood state among adolescents in the pandemic.
	**Qi et al**. **(****12****)**. https://doi.org/10.1016/j.jadohealth.2020.07.001	China, Asia	“To explore the association between the levels of social support and mental health among Chinese adolescents during the outbreak.”	Social support	Depression and anxiety	There was a higher prevalence of mental health problems among adolescents with medium and low levels of social support
	**Ren et al**. **(****13****)**. https://doi.org/10.1016/j.jadohealth.2020.09.026	China, Asia	“To identify potential protective factors that may buffer the association between the presence of COVID-19 cases in adolescents' communities and their post-quarantine depressive symptoms.”	COVID-19 cases in community	Depression	The presence of COVID-19 cases in communities contributed to adolescents' poorer mental health, and the association was stronger for older adolescents.
	**Shuang-Jiang et al**. **(****14****)**. https://doi.org/10.1007/s00787-020-01541-4	China, Asia	“To assess the prevalence of two specific mental symptoms, anxiety, and depression, and their socio-demographic correlates among adolescents in the Chinese population during the COVID-19 outbreak.”	COVID-19 pandemic	Anxiety and depression	The prevalence of depressive symptoms, anxiety symptoms, and a combination of depressive and anxiety symptoms was 44, 37, and 31%, respectively. There was a high prevalence of psychological health problems among adolescents, which was negatively associated with the level of knowledge about and the prevention and control measures for COVID-19
	**Tang et al**. **(****15****)**. https://doi.org/10.1016/j.jad.2020.10.016	China, Asia	“To estimate the prevalence of depressive, anxiety, and stress symptoms, and levels of life satisfaction, among children and adolescents experiencing home quarantine and school closure in Shanghai due to COVID-19.”	Home quarantine and school closure	Depression, anxiety, and stress	25% had experienced symptoms of anxiety, followed by 20% for depressive symptoms and 15% for stress symptoms. 12% met threshold for depression, anxiety, and stress altogether. Participants were generally satisfied with life and 21% became more satisfied with life during school closures.
	**Tso et al**. **(****16****)**. https://doi.org/10.1007/s00787-020-01680-8	China, Asia	“To investigate and identify the characteristics of children vulnerable to the negative impacts of the COVID-19 pandemic.”	COVID-19 pandemic, school closures and containment measures	Parent–child interaction, emotional and behavioural problems, quality of life	Compared to reference means, children demonstrated significantly more psychosocial problems, fewer prosocial behaviours, and poorer functioning.
	**Yang et al**. **(****17****)**. https://doi.org/10.1016/j.childyouth.2020.105634	China, Asia	“To explore the psychological influence of COVID-19 on Wuhan's adolescents and verified the mediation effects of resilience and positive emotion regulation on the relationship between psychological trauma and mental health.”	COVID-19 pandemic, resilience and positive emotion regulation	Psychological trauma and mental health	Resilience and positive emotion regulation interrupted the direct impact of psychological trauma on mental health, thereby greatly protecting mental health
	**Yue et al**. **(****18****)**. https://doi.org/10.1007/s12144-020-01191-4	China, Asia	“To examine the psychological status among families in China facing the COVID-19 outbreak and the associated risk and positive factors.”	COVID-19 pandemic	Anxiety, depression and post-traumatic stress disorder (PTSD)	2% experienced moderate anxiety, 2% experienced depression and 3% met the diagnostic criteria for PTSD. Excessive media exposure was a risk factor for anxiety and PTSD in children.
	**Zhang et al**. **(****19****)**. https://doi.org/10.1016/j.jadohealth.2020.08.026	China, Asia	“To assess the psychological impacts of the COVID-19 pandemic on junior high and high school students.”	COVID-19 pandemic	Resilience, coping, depression, post-traumatic stress disorder (PTSD)	Moderate depressive symptoms were found in 9% of junior school and 7% of high school students, and severe to extremely severe depressive symptoms were found in 5% of junior school and 3% of high school students. Moderate anxiety symptoms were found in 10% of junior school and 11% of high school students, and severe to extremely severe anxiety symptoms were found in 10% of junior school and 7% of high school students. Moderate stress symptoms were found in 6% of junior high school students and 7% of high school students, and severe to extremely severe stress symptoms were found in 3% of junior school and 3% of high school students. Trauma-related distress was found in 21% of junior school and 23% of high school students, with no significant between-group differences.
	**Zhang et al**. **(****20****)**. https://doi.org/10.12659/MSM.924994	China, Asia	“To explore the emotional resilience of middle school students from during an ongoing pandemic and assessed its influence on students' learning management skills.”	COVID-19 pandemic	Emotional resilience and learning management	Emotional resilience was positively correlated with learning management skills, and positive emotional ability predicted learning management skills.
	**Zhang et al**. **(****21****)**. https://doi.org/10.3390/ijerph17207666	China, Asia	“To examine the impacts of social isolation on PA levels and mood states of children and adolescents and to explore the correlation between them during the COVID-19 epidemic.”	Social isolation and physical activity levels	Mood state	Physical activity level had a significantly positive impact on the mood states of children and adolescents during the COVID-19 pandemic
	**Zhou et al**. **(****22****)**. https://doi.org/10.1186/s12992-020-00601-3	China, Asia	“To determine the incidence and correlates of depressive symptoms among female adolescents during the COVID-19 outbreak in mainland China.”	COVID-19 pandemic	Depression	40% of female adolescents met threshold for depression
	**Darvishi et al**. **(****23****)**. https://doi.org/10.1007/s41470-020-00077-x	Iran, Asia	“To evaluate the prevalence of obsessive-compulsive disorder (OCD) and cognitive errors among young people during the outbreak of coronavirus disease 2019.”	COVID-19 pandemic	OCD	OCD symptomatology was reported by 67% of adolescents. The highest prevalence of obsessive-compulsive disorder symptom belonged to washing compulsion.
	**Fazeli et al**. **(****24****)**. https://doi.org/10.1016/j.abrep.2020.100307	Iran, Asia	“To examine the mediating role of psychological distress (depression, anxiety, and stress) in the association between internet gaming disorder and two health outcomes (insomnia, quality of life) among adolescents during this COVID-19 pandemic.”	Internet/technology use	Depression, anxiety, stress, insomnia, quality of life	There was a mediating effect of depression, anxiety, and stress on the associations between internet gaming disorder and insomnia, adolescent-reported quality of life, and parent-reported quality of life.
	**Shorer and Leibovich (****25****)**. https://doi.org/10.1080/03004430.2020.1806830	Israel, Asia	“To explore young children's emotional adjustment during the COVID-19 outbreak as it relates to their exposure to stress, and their parents' emotion regulation and playfulness.”	COVID-19 pandemic, parental emotion regulation and playfulness	Stress	The most frequent stress symptoms in children were nervousness, agitation, aggression, separation fears and clinging. Parental difficulties in emotion regulation, and the level of exposure to stressogenic situations were both significantly associated with children's stress reactions. Parental emotion regulation fully mediated the relationship between exposure to stress and children's stress reactions.
	**Lin (****26****)**. https://doi.org/10.3390/ijerph17228547	Taiwan, Asia	“To examine the prevalence of Internet addiction and identify the psychosocial risk factors during the COVID-19 outbreak.”	Internet/technology use	Well-being, depression, neuroticism, impulsivity, self-esteem	Impulsivity was positively related to Internet addiction.
	**Adibelli and Sümen (****27****)**. https://doi.org/10.1016/j.childyouth.2020.105595	Turkey, Asia/Europe	“To examine the effect of the COVID-19 pandemic on health-related quality of life in children.”	COVID-19 pandemic	Quality of life	Self-reported quality of life scores of children were generally good. The highest average score was for “physical well-being” and “family,” while the lowest average score was for “friends” and for “self-esteem.”
	**Kilinçel et al**. **(****28****)**. https://doi.org/10.1111/appy.12406	Turkey, Asia/Europe	“To determine the results of home-quarantine measures taken for adolescents during the pandemic and the factors affecting these results.”	Home quarantine measures	Loneliness and anxiety	The closure of schools and home-quarantine increased levels of anxiety and loneliness. Exposure to excessive information caused elevated levels of stress and anxiety among children.
	**Seçer and Ulaş (****29****)**. https://doi.org/10.1007/s11469-020-00322-z	Turkey, Asia/Europe	“To examine the mediating role of emotional reactivity, depression anxiety and experiential avoidance in the relationship between the fear of COVID-19 and obsessive compulsive disorder (OCD) symptoms in adolescents.”	Fear of COVID-19	OCD, emotional reactivity, avoidance, depression, and anxiety	The effect of COVID-19 fear on OCD is mediated by emotional reactivity, experiential avoidance and depression-anxiety
**Africa**	**Rakhmanov et al**. **(****30****)**. https://www.jrmds.in/articles/the-effects-of-covid19-pandemic-on-anxiety-in-secondary-school-students.pdf	Nigeria, Africa	“To investigate the effects of COVID-19 pandemic on anxiety levels in Nigerian secondary school students.”	COVID-19 pandemic and lockdown	Anxiety	Isolation had no statistically significant effects on COVID-19 anxiety. Examination anxiety was lower in an isolated group compared with a non-isolated group.
**Europe**	**Cauberghe et al**. **(****31****)**. https://doi.org/10.1089/cyber.2020.0478	Belgium, Europe	“To examine if social media are beneficial for adolescents to cope with feelings of anxiety and loneliness during the quarantine.”	Internet/technology use	Loneliness, anxiety, and depression	Anxious participants indicated they used social media more often to actively seek for a manner to adapt to the current situation, and to a lesser extent as a way to keep in touch with friends and family. Participants who were feeling lonely were more inclined to use social media to cope with lacking social contact.
	**Crescentini et al**. **(****32****)**. https://doi.org/10.3389/fpsyg.2020.586074	Italy, Europe	“To investigate the immediate psychological effects of COVID-19 pandemic and the consequent lockdown on children, as reported by their parents, and on parents themselves (among others).”	COVID-19 pandemic and lockdown	Distress/post-traumatic stress disorder, child behaviour, anxiety, and depression	Internalising symptoms of parents and children were significantly higher during the COVID-19 pandemic than before it started.
	**Cusinato et al**. **(****33****)**. https://doi.org/10.3390/ijerph17228297	Italy, Europe	“To investigate parents' and children's well-being, parental stress, and children's resilience during the COVID-19 pandemic, more specifically during the quarantine (among others).”	COVID-19 pandemic and quarantine	Well-being and resilience	Confinement measures and changes in daily routine negatively affected both children's and parents' behavioural and emotional dimensions. Some parents (18%) reported negative effects of the confinement measures on their interactions with their children, whereas the majority (49%) reported positive changes in the parent-child relationship (e.g., spending more time together).
	**di Cagno et al**. **(****34****)**. https://doi.org/10.3390/ijerph17238867	Italy, Europe	“To evaluate the acute stress-perception and stress-response reactions to sports activity interruption, due to the quarantine measures.”	Quarantine and sports interruption	Psychological distress level	More than 50% of child athletes and 32% of adolescent athletes scored at or above threshold on the Impact of Event scale, indicating perceived psychological distress.
	**Di Giorgio et al**. **(****35****)**. https://doi.org/10.1007/s00787-020-01631-3	Italy, Europe	“To characterising the changes in mothers' and children' sleep quality, subjective time experience, emotional symptoms, and self-regulation capacity during the lockdown compared to the period immediately before.”	Lockdown (restrictive measures)	Well-being, emotional and behavioural problems	Children showed increased boredom and difficulties to follow daily routines. The proportion of children with self-control difficulties increased from 14% before to 21% during the lockdown. An increase in emotion symptoms, conduct problems, and hyperactivity/ inattention issues were reported during the lockdown, regardless of the mother-working situation.
	**Morelli et al**. **(****36****)**. https://doi.org/10.3389/fpsyg.2020.584645	Italy, Europe	“To investigate parental correlates of children's emotion regulation during the COVID-19 lockdown.”	COVID-19 pandemic, parental self-efficacy	Emotion regulation	Parents' beliefs to be competent in managing parental tasks might be a protective factor for their children's emotional well-being.
	**Spinelli et al**. **(****37****)**. https://doi.org/10.3389/fpsyg.2020.01713	Italy, Europe	“To explore the effect of risk factors associated with the COVID-19 outbreak experience on parents' and children's well-being.”	Quarantine-related risk factors	Emotional and behavioural problems	Overall there were no relevant associations of COVID-19 contact risk index and home environment risk index with dyadic parenting stress, parental individual stress, and child emotional and behavioural problems. Perception of the difficulty of quarantine was related to both parents' and children's well-being. The impact of quarantine on children's emotional and behavioural problems was mediated by parental individual and dyadic stress.
	**Orgilés et al**. **(****38****)**. https://doi.org/10.31234/osf.io/5bpfz	Italy and Spain, Europe	“To examine the emotional impact of the quarantine on children and adolescents.”	COVID-19 pandemic, quarantine	Emotional state	86% of parents perceived changes in their children's emotional state and behaviours during the quarantine. The most frequent symptoms were difficulty concentrating (77%), boredom (52%), irritability (39%), restlessness (39%), nervousness (38%), feelings of loneliness (31%), uneasiness (30%), and worries (30%). 11% reported family coexistence during quarantine was “difficult” or “very difficult,” and 62% “easy” or “very easy.”
	**Ezpeleta et al**. **(****39****)**. https://doi.org/10.3390/ijerph17197327	Spain, Europe	“To explore if life conditions of adolescents during lockdown are associated with mental health problems.”	Lockdown life conditions	Emotional and behavioural problems	Conduct, peer, prosocial, and total problems scores increased after lockdown.
	**Romero et al**. **(****40****)**. https://doi.org/10.3390/ijerph17196975	Spain, Europe	“To examine the effects of the Spanish confinement derived from the COVID-19 crisis on children and their families.”	COVID-19 pandemic, confinement	Emotional and behavioural problems	A majority of children did not show any change in behaviours addressing negative outcomes (i.e., conduct problems, emotional problems and hyperactive behaviour). However, a higher proportion of children increased rather than decreased negative outcomes, particularly for hyperactivity.
**North America**	**Carroll et al**. **(****41****)**. https://doi.org/10.3390/nu12082352	Canada, North America	“To examine how health behaviours and level of family stress, financial and food security have changed from before/during COVID-19.”	COVID-19 pandemic	Stress	According to parents, almost half (49%) of children had very little concern about COVID-19, 38% were somewhat concerned, and 7% were very much concerned.
	**Ellis et al**. **(****42****)**. http://dx.doi.org/10.1037/cbs0000215	Canada, North America	“To examine the relationships between psychological adjustment and reported stress associated with the initial COVID-19 crisis.”	COVID-19 pandemic	Stress, loneliness, and depression	43% reported to be “very concerned” about the pandemic. As a whole, adolescents' stress about COVID-19 was significantly related to poorer adjustment, including more reported depression and greater loneliness.
	**Drouin et al**. **(****43****)**. https://doi.org/10.1089/cyber.2020.0284	United States, North America	“To examine the interaction between social media use and feelings of anxiety during times of crisis.”	Internet/technology use	Anxiety	Moderate or severe anxiety symptoms were reported by 50% of parents and 63% rated their child as experiencing anxiety symptoms on several days or more. 86% felt that social distancing restrictions had at least a small negative effect on their child's mental health.
	**Fitzpatrick et al**. **(****44****)**. https://doi.org/10.1007/s10578-020-01089-z	United States, North America	“To identify the primary mental health problems and needs of children, adolescents, and their caregivers during COVID-19.”	COVID-19 pandemic	Depression, anxiety	Children's depression and anxiety symptom means fell within the clinical range. Mental health services were ranked the most urgent for caregivers and adolescents, second for 6–12 year olds, and third for 1–5 year olds.
	**Gassman-Pines et al**. **(****45****)**. https://doi.org/10.1542/peds.2020-007294	United States, North America	“To understand whether and how the COVID-19 crisis has affected parents' and children's psychological well-being.”	COVID-19 pandemic	Well-being	Many families have experienced hardships during the crisis, including job loss, income loss, caregiving burden, and illness.
	**Oosterhoff et al**. **(****46****)**. https://doi.org/10.1016/j.jadohealth.2020.05.004	United States, North America	“To examine adolescents' motivations and engagement in social distancing, and their mental and social health.”	Social distancing	Anxiety, depression, belonging, burden	No evidence of an association between degree of social distancing engagement and any indicator of mental or social health.
	**Patrick et al**. **(****47****)**. https://doi.org/10.1542/peds.2020-016824	United States, North America	“To determine how the pandemic and mitigation efforts affected the physical and emotional well-being of parents and children.”	COVID-19 pandemic and mitigation efforts	Well-being	14% of the parents reported worsening behavioural health for their children.
	**Russell et al**. **(****48****)**. https://doi.org/10.1007/s10578-020-01037-x	United States, North America	“To examine concurrent patterns of parents' experience from a national sample during the early months of the U.S. COVID-19 pandemic.”	COVID-19 pandemic	Stress and child-parent relationship	Results indicate significant linkages between parents' caregiver burden, mental health, and perceptions of children's stress; these in turn are significantly linked to child-parent closeness and conflict.
**South America**	**Garcia de Avila et al**. **(****49****)**. https://doi.org/10.3390/ijerph17165757	Brazil, South America	“To assess the prevalence of anxiety among Brazilian schoolchildren and study the anxiety factors associated with social distancing during the global COVID-19 pandemic.”	COVID-19 pandemic	Anxiety	The prevalence of anxiety among children (22%) was high compared to previous research.
	**Asanov et al**. **(****50****)**. https://doi.org/10.1016/j.worlddev.2020.105225	Ecuador, South America	“To learn how students spend their time during the period of quarantine, examine their access to remote learning, and measure their mental health status.”	Remote learning and quarantine	Depression	Closure of schools and social isolation were the two main problems students say they face. Whilst the majority (68%) were “happy,” 16% of students had mental health scores that indicate major depression.

**Table 3:** Overview of longitudinal studies organised by continent and country.

	**Author, year, title, DOI**	**Country, continent**	**Tools utilised in study**	**Outcomes relevant to the review**
**Asia**	**Chen et al**. **(****51****)** Problematic internet-related behaviors mediate the associations between levels of internet engagement and distress among schoolchildren during COVID-19 lockdown: a longitudinal structural equation modeling study. https://doi.org/10.1556/2006.2021.00006	China, Asia	**Problematic smartphone-application use:** Smartphone Application-Based Addiction Scale (SABAS): addiction components model. **Problematic social media use:** Bergen Social Media Addiction Scale (BSMAS) **Internet gaming disorder (IGD):** Internet Gaming Disorder Scale-Short Form (IGDS-SF9) **Psychological distress:** Depression, Anxiety, Stress Scale-21 (DASS-21)	School children had greater psychological distress during the COVID-19 school suspension compared with before the COVID-19 outbreak School children spent significantly more time on smartphones and social media but not gaming during the school suspension compared to baseline. Increased problematic use of internet-related activities among school children was associated with greater psychological distress.
	**Teng et al**. **(****52****)** Depression and anxiety symptoms associated with internet gaming disorder before and during the COVID-19 pandemic: a longitudinal study. https://doi.org/10.1556/2006.2021.00016	China, Asia	**Videogame use:** open questions where participants list the names of their three favourite videogames, indicating how frequently they played each game **Internet Gaming Disorder (IDG)**: The Internet Gaming Disorder Scale-Short Form (IGDS9-SF) **Degree of perceived impacts caused by the COVID-19 pandemic on different life domains**: Perceived COVID-19 impacts **Depression**: Chinese version of the Centre for Epidemiologic Studies Depression Scale(CES-D) **Anxiety**: The Chinese version of the State-Trait Anxiety In-ventory (STAI)	For anxiety symptoms, the results showed significantly higher scores at T2 in comparison to T1 in the total sample and adolescent sample but not in the child sample. With regards to depressive symptoms, no significant differences emerged across any of the samples. Both videogame use and IGD increased significantly for adolescents at T2. Depressive and anxiety symptoms at T1 positively predicted IGD and video game use at T2 (especially for boys), but not inversely. Perceived COVID-19 impacts mediated the relationship between depressive and anxiety symptoms at T1 and IGD at T2.
**Europe**	**Paschke et al**. **(****53****)** Risk factors for prospective increase in psychological stress during COVID-19 lockdown in a representative sample of adolescents and their parents. https://doi.org/10.1192/bjo.2021.49	Germany, Europe	**Confidence in parenting:** Parental Self-efficacy Questionnaire **Adolescents' and parents' emotion regulation problems**: Difficulties in Emotion Regulation Scale, short form **Behavioural avoidance**: Procrastination Questionnaire for Students **Change in psychological stress:** Perceived Stress Scale (PSS-4)	35% of adolescents reported a significant increase in psychological stress. Adolescents and parents who experienced a significant increase in psychological stress had lower baseline stress levels than those who did not experience increased psychological stress during COVID-19 lockdown. Significant adolescent risk factors for increased psychological stress included financial worries, increased psychological stress of the corresponding parent, procrastination, limited access to emotion regulation strategies and mainly staying at home during COVID-19 lockdown. High emotional awareness served as a protective factor for adolescents.
	**Alivernini et al**. **(****54****)** Physical distancing behavior: the role of emotions, personality, motivations, and moral decision-making. https://doi.org/10.1093/jpepsy/jsaa122	Italy, Europe	**Positive and Negative Affect:** Positive and Negative Affect Schedule – Children (PANAS-C) **Personality Traits**: Italian Ten-Item Personality Inventory (I-TIPI) **Motivation to Engage in PDB**: Autonomous motivation (e.g., “I engage in PDB because I find it personally meaningful”) and controlled motivation (e.g., “I engage in PDB because I would feel ashamed not to”). **Moral Disengagement**: assessed by six items (adapted from a previous survey on Italian young people) **Intention to Engage in Physical Distancing Behaviour (PDB)**: assessed by six items (adapted from a previous survey on Italian young people) **Physical Distancing Behaviour**: assessed by three items	Compared with 1 year earlier, adolescents experienced fewer positive emotions and more negative emotions after the COVID-19 national lockdown
	**Giménez-Das**í **et al**. **(****55****)** Six weeks of confinement: psychological effects on a sample of children in early childhood and primary education. https://doi.org/10.3389/fpsyg.2020.590463	Spain, Europe	**System of Evaluation of Children and Adolescents (SENA) (**selected scales): Attentional Problems; Depression, Challenging Behaviours; Emotional regulation; Hyperactivity; Willingness to study (only for the Primary Education group)	No differences in mean scores before and after 4–6 weeks of confinement were detected among the 3-year-old children. Some change was observed among the 6–10-year-olds. The children obtained lower scores in dimensions related to self-regulation (emotional, attentional, and behavioural) and in willingness to study. No change was identified for depression or challenging behaviour
	**Achterberg et al**. **(****56****)** Perceived stress as mediator for longitudinal effects of the COVID-19 lockdown on wellbeing of parents and children. https://doi.org/10.1038/s41598-021-81720-8	The Netherlands, Europe	**Children's externalising and internalising behaviour**: Strength and Difficulties Questionnaire (SDQ), shortened version **Covid-19 lockdown:** Bespoke questions on COVID-19 lockdown related aspects **Perceived stress:** Perceived Stress Scale (PSS) **Coping strategies:** Positive and negative coping strategies	Decrease in externalising behaviour over time across development, and this decrease is decelerated by the COVID-19 pandemic lockdown. There was no significant influence of the COVID-19 lockdown on children's internalising behaviour. 21% of the children indicated that stress applied to them in the last 2 weeks of the COVID-19 lockdown. Children's externalising behaviour during the lockdown was significantly predicted by prior externalising behaviour. Children with higher levels of externalising behaviour prior to the lockdown perceived more stress during the lockdown, resulting in an increase in externalising behaviour during the lockdown. Perceived stress of children and parents were not significantly correlated. Parental over-reactivity was significantly related to children's perceived stress.
	**Janssen et al**. **(****57****)** Does the COVID-19 pandemic impact parents' and adolescents' well-being? An EMA-study on daily affect and parenting. https://doi.org/10.1371/journal.pone.0240962	The Netherlands, Europe	**Ecological Momentary Assessment measures:** Affect: adapted and shortened four-item version of the Positive and Negative Affect Schedule for Children (PANAS-C); Daily parenting and Daily difficulties and helpful activities: participants were asked to choose items from a list of potential activities **Intolerance of uncertainty:** 12-item version of the Intolerance of Uncertainty Scale (IUS) **Depressive symptom**s: Patient Health Questionnaire (PHQ-9)	Adolescents reported 5 main difficulties: boredom (23%), missing social contact with friends (18%), irritations with family members (13%), homework (12%), and worry about the health of others (6%). No differences in adolescents' negative affect nor positive affect during the COVID-19 pandemic, as compared to a baseline period, were reported. Parental warmth and criticism, from both the parent and adolescent perspective, did not differ between before and during the COVID-19 pandemic. Intolerance of uncertainty was linked to greater negative affect in adolescents, and linked to a decrease in adolescents' positive affect.
	**Bignardi et al**. **(****58****)** Longitudinal increases in childhood depression symptoms during the COVID-19 lockdown. http://dx.doi.org/10.1136/archdischild-2020-320372	United Kingdom, Europe	**Emotional Problems:** Strengths and Difficulties Questionnaire (SDQ), emotional problems subscale **Anxiety and Depression:** Revised Child Anxiety and Depression Scale (RCADS) short form **Neighbourhood deprivation:** Index of Multiple Deprivation	A significant increase in depression symptoms during the UK lockdown was found. Changes in anxiety and emotional problems were small and not statistically significant.
**North America**	**Browne et al**. **(****59****)** Children's mental health problems during the initial emergence of COVID-19. https://doi.org/10.1037/cap0000273	Canada, North America	**Emotional and Behavioural Problems:** Strengths and Difficulties Questionnaire (SDQ), total difficulties scale **Impairment:** The Impairment Rating Scale (IRS)	Male children enrolled in early childhood education showed a modest decline in mental health problems prior to the pandemic announcement by the WHO. However, following the WHO announcement, male children's mental health problems worsened significantly. No post-pandemic differences over time were observed for females.
	**Hawes et al**. **(****60****)** Increases in depression and anxiety symptoms in adolescents and young adults during the COVID-19 pandemic. https://doi.org/10.1017/S0033291720005358	United States, North America	**Depression:** Children's depression inventory (CDI) **Anxiety**: Screen for child anxiety-related disorders (SCARED) **Experiences related to the COVID-19 pandemic**: Pandemic experiences survey	Psychiatric symptoms increased during the pandemic, including depression, panic/somatic symptoms, generalised anxiety, and social anxiety. *Post-hoc* analyses reveal that depression and panic/somatic symptoms increased for females. In female participants, there was a nearly 3-fold increase in rates of clinically elevated depression from before to during the pandemic and nearly half (49%) experienced clinically elevated generalised anxiety during the pandemic. Greater COVID-19 school concerns were associated with increased depression symptoms and greater COVID-19 home confinement concerns were associated with increased generalised anxiety symptoms, and decreased social anxiety symptoms. None of the composites were associated with panic/somatic symptoms. Gender did not moderate any relationship between pandemic experiences and change in CDI or SCARED symptoms.
	**Hawes et al**. **(****61****)** Trajectories of depression, anxiety and pandemic experiences; A longitudinal study of youth in New York during the Spring-Summer of 2020. https://doi.org/10.1016/j.psychres.2021.113778	United States, North America	**Depression:** Children's Depression Inventory (CDI) **Anxiety:** Screen for Child Anxiety Related Disorders (SCARED) **Pandemic experiences:** Pandemic experiences survey	Multilevel growth modelling indicated that symptoms of depression and anxiety peaked around late April/early May and then decreased through May–July. Some pandemic experiences followed a similar quadratic trajectory, while others decreased linearly across the study. Specific relationships emerged between some types of pandemic experiences and depression and anxiety symptoms.
	**Penner et al**. **(****62****)** Change in youth mental health during the COVID-19 pandemic in a majority Hispanic/Latinx US sample. https://doi.org/10.1016/j.jaac.2020.12.027	United States, North America	**Effects of the COVID-19 Pandemic at Home:** COVID-19 survey for youths, adapted: experiences at home during the COVID-19 pandemic **Emotional and behavioural problems**: Brief Problem Monitor (BPM)	The majority of youths reported only “a little” or “not at all” for difficult family relationships, loneliness, stress, parent stress, conflict with parents, worsened relationships with parents, and parent impatience during the pandemic. Around 80% of students reported “a little,” “a lot,” or “a whole lot” for parents' level of understanding, ability to make the child feel better, and ability to help the child manage stress during the pandemic. For youths who had elevated levels of mental health problems before the pandemic, symptoms were significantly reduced across domains during the pandemic. Reductions in internalising, externalising, and total problems were clinically significant. For other youths, there were statistically significant reductions in internalising and total problems, and no change in attention or externalising problems. *Post hoc* analyses revealed that better family functioning was consistently related to lower mental health symptoms in youths during COVID-19 follow-ups.
	**Rogers et al**. **(****63****)** Adolescents' perceived socio-emotional impact of COVID-19 and implications for mental health: results from a U.S.-based mixed-methods study. https://doi.org/10.1016/j.jadohealth.2020.09.039	United States, North America	**Indices of mental health:** Depressive symptoms: the Children's Depression Inventory short version; Anxiety symptoms: the seven-item Generalised Anxiety Disorder Scale and Loneliness: was assessed using the three-item Loneliness Scale **Perceived relationship changes during COVID-19 pandemic:** Six questions were developed to assess how relationships had changed during the COVID-19 pandemic **Perceived mood changes during COVID-19 pandemic:** Six items were developed regarding mood changes during COVID-19	In general adolescents reported low levels of mental health problems before the COVID-19 pandemic. Small significant increases in depressive symptoms, anxiety symptoms and loneliness were detected between before and during the pandemic. Adolescents reportedly spent less time with friends and, despite online interaction, felt a lack of emotional connexion and a decrease in overall friend support. On the contrary, adolescents perceived overall increases in family support and some adolescents perceived decreases in family conflict during COVID-19. The decline in friend support during the pandemic was found to be related to higher depressive symptoms, and the feeling of more conflict with friends during the pandemic was also found to be related to more loneliness. Additionally, greater perceived increases in family conflict during the pandemic was found to be related to more depressive symptoms and greater loneliness.
**Australia**	**Magson et al**. **(****64****)** Risk and protective factors for prospective changes in adolescent mental health during the COVID-19 pandemic. https://doi.org/10.1007/s10964-020-01332-9	Australia, Australia	**Generalised anxiety:** Generalised Anxiety subscale of the Spence Children's Anxiety Scale **Depressive symptoms**: Short Mood and Feelings Questionnaire - Child Version **Life satisfaction**: Student's Life Satisfaction Scale **COVID-19 related distress:** 18 items were developed to assess COVID-19 related distress **Disruption to schooling:** four items were developed to assess format of school attendance, difficulties during online learning, motivation to complete school work, and impact on education **Media exposure:** two items to assess exposure to traditional news media **Interpersonal conflict:** four items were developed to assess change in interpersonal conflict between adolescents and their mothers, fathers, siblings, and friends due to the COVID-19 social distancing rules and stay at home restriction **Social connectedness:** Social Connectedness Scale **Adherence to COVID-19 Australian government stay-at home directive:** how often adolescents had left their home	In general, adolescents reported low to moderate levels of COVID-19 related stress. The most distressing issue reported was not being able to see friends, closely followed by a friend or family member contracting and getting very sick and/or dying from COVID-19. Significant increases in depression and anxiety symptoms were reported as well as a significant decrease in life satisfaction from before to during the pandemic, which was particularly pronounced among girls. Age did not moderate change in depressive symptoms, anxiety, or life satisfaction. COVID-19 related stress, online learning difficulties, and increased conflict with parents predicted increases in mental health problems from before to during the pandemic, whereas adherence to stay-at-home orders (government restrictions) and feeling socially connected (greater exposure to traditional media) during the COVID-19 lockdown was associated to less distress.
	**Munasinghe et al**. **(****65****)** The impact of physical distancing policies during the COVID-19 pandemic on health and well-being among Australian adolescents. https://doi.org/10.1016/j.jadohealth.2020.08.008	Australia, Australia	**Psychological well-being:** Psychological distress: Kessler Psychological Distress 6-item scale; Well-being: The Engagement, Perseverance, Optimism, Connectedness, and Happiness (EPOCH); Social relationships: measured based on the question: “In the past hour, who were you with?” **Physical Activity:** PACE + Adolescent Physical Activity Measures **Sedentary behaviour:** Adolescent Sedentary Activities Questions (modified) **Diet:** New South Wales Centre for Public Health Nutrition	Physical distancing measures were associated with decreases in happiness and positive emotions and slightly increased psychological distress.

## Results

**Figure 1:**
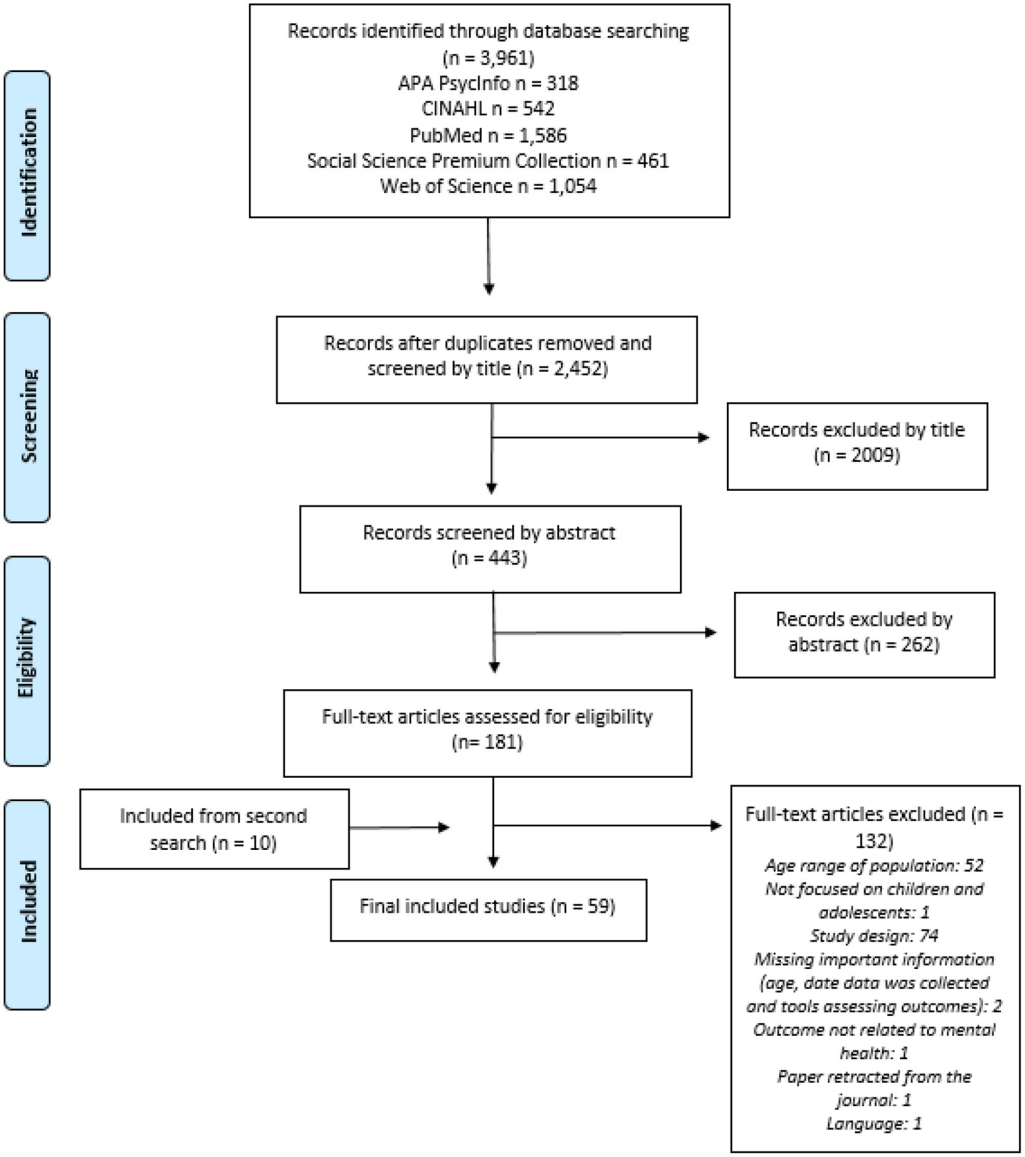
Flow diagram of the selection process.

The first search yielded 2,452 non-duplicate references and resulted in 49 eligible studies (*longitudinal and cross sectional*) while the second search, used to update the review, yielded 3,309 non-duplicate references and resulted in 10 eligible studies (*longitudinal only*) (Figure 1). Some included studies had multiple aims, not all of which were related to a mental health outcome; only findings related to mental health outcomes were reported in this review.

### Has the COVID-19 Pandemic and Societal Infection Control Measures Impacted Child and Adolescent Mental Health?

The review identified many cross-sectional studies investigating mental health symptomatology among children and adolescents during the pandemic period (Table 2). When compared with average scores prior to the pandemic, significantly increased levels of psychosocial problems were reported across international studies (16, 32, 35, 39, 49). There were also accounts of adolescents (42) and parents (38) directly reflecting on the impact of COVID-19 on mental health, which indicate concern. One study in China made a geographical comparison, demonstrating significantly higher levels of anxiety symptomatology among adolescents in the COVID-19 outbreak region of Wuhan compared with other urban areas (8). Reports were most common on symptoms of anxiety (8–10, 12, 14, 15, 18, 24, 28–32, 43, 44, 46, 49) and depression (8–10, 12–15, 18, 19, 22, 24, 26, 29, 31, 32, 42, 44, 46, 50), but other mental health disorders such as obsessive compulsive disorder (23, 29) and post-traumatic stress disorder (17–19, 25, 32) have been investigated as well as stress (9, 15, 24, 25, 34, 41, 42, 48), loneliness (28, 31, 42), and well-being (26, 33, 35, 45, 47) among other outcomes (Table 2). Although most of these studies indicate raised levels of mental health concerns among children and adolescents during the pandemic period, the evidence is mixed with some reporting no behavioural changes (40), good levels of well-being (27, 50) or even suggestion of improvement (15).

Yet, cross-sectional studies are descriptive in nature and it is not possible to infer causality from this research design. The scoping review discovered 15 longitudinal studies (Table 3), which involved repeated measures over time and provide stronger evidence to address the question of impact on mental health. Five longitudinal studies involved children (51, 55, 56, 58, 59), nine involved adolescents (53, 54, 57, 60–65) and one involved children and adolescents (52). Most of the studies indicated negative impact of the pandemic on mental health, including increased symptoms of depression (60, 63, 64, 66), anxiety (60, 63, 64, 67), loneliness (63), psychological distress (51, 53, 65), hyperactivity and impulsivity (55), and emotional and behavioural problems (59), as well as reductions in emotional regulation (55), happiness and positive emotions (54, 65), and life satisfaction (64). However, a study from Spain reported no significant change among preschool-aged children and, despite some statistically significant differences in a primary school-aged group, no change was identified for depression or challenging behaviour (55). Similarly, a study from China reported significant changes in anxiety among adolescents but not children, and no change was identified for depression (67). In a Canadian study, significant impact on emotional and behavioural problems was detected for male children enrolled in early childhood education, but not females (59). A study from Australia reported no differences in adolescents' reports on negative affect, nor positive affect, during the pandemic as compared to a baseline period (57). Longitudinal research from the Netherlands reported findings with a developmental perspective; although a slight reduction was seen in externalising problems from pre-pandemic to during pandemic, this was considered in line with developmental trajectory and it was interpreted that the pandemic had decelerated the expected reduction (56). A further study, which used a majority Hispanic/Latinx sample in the United States (US), reported a reduction in emotional and behavioural problems from before to during the pandemic; the reduction was greater for those who had elevated mental health problems pre-pandemic (62).

One of the identified longitudinal studies did not seek to compare pre-and post-pandemic mental health outcomes but instead to track the trajectory of mental health symptomatology during the pandemic period among youth in New York, US (68). This study reported that symptoms of depression and anxiety peaked around late April/early May and then decreased through May to July 2020 (68).

When specifically considering the impact of societal control measures, which have varied internationally, many studies suggested negative impact yet the evidence was mixed. The closure of schools and home quarantine, sometimes referred to as “lockdown,” was reportedly negatively associated with children's mental health outcomes across various international settings (28, 33, 39, 53, 54, 56, 66), as were physical distancing measures (65). Boredom and difficulty concentrating emerged as specific concerns (35, 38), which is understandable given the loss of routine that comes with such control measures. In a couple of studies, the children themselves reported the closure of schools, social isolation and not being able to see friends as the most pressing problems they were facing during the pandemic (50, 64), and a further study reported that decline in support from friends was associated with higher depressive symptoms (63). Yet, other studies reported a lack of association between degree of social distancing engagement (46) and isolation (30) with mental health outcomes, and one study even detected an association between adherence to stay-at-home orders and *lower* levels of distress (64). Interestingly, one study indicated that greater COVID-19 home confinement concerns were associated with *increased* generalised anxiety symptoms, yet *decreased* social anxiety symptoms (60). There was also indication of the societal control measures enhancing family togetherness (33, 38, 63), and in one study around a fifth of the children reported being *more* satisfied with life during school closures (15).

### What Is the Evidence From Different Geographical Regions?

Nearly half of the included studies were conducted in Asia (*n* = 25), predominantly in China (*n* = 17/25). As COVID-19 originated in China, it is not surprising that the country is at the forefront of publishing research about the pandemic; yet, it must be recognised that many factors affect investigation time frames. The Chinese government imposed strict containment measures in January 2020, which were eased from February 2020 with localised restrictions re-imposed in new “hotspots.” The number of reported COVID-19 cases has remained low ever since. Although relatively brief, the societal infection control measures in China were among the strictest worldwide. Theoretically, it could be argued that such strict measures had a particularly adverse effect on children and adolescents' mental health. Most of the Chinese research was cross-sectional (*n* = 15/17), and largely explored depression and/or anxiety symptomatology among children and adolescents (*n* = 10/15), with prevalence rates ranging from 2 to 44%. Despite the mixed cross-sectional evidence, the two longitudinal studies conducted in China (51, 67) reported increased psychological distress, particularly anxiety symptoms among adolescents.

Further research from Asia was conducted in Bangladesh (*n* = 1), Iran (*n* = 2), Israel (*n* = 1), Taiwan (*n* = 1), and Turkey (*n* = 3). All of these studies were cross-sectional. Although some studies only reported prevalence of mental health symptomatology (23) and perceived quality of life (27), others explored mediating factors and indicated evidence of interaction between internet-related behaviours and child and adolescent mental health (24, 26), as well as parental mental health and child and adolescent mental health (7, 25).

Only one included study was from Africa, which was conducted in Nigeria where a nationwide lockdown was introduced in late March 2020 and lifted in May 2020 due to public unrest about the socio-economic consequences. This single study explored the impact of isolation on school students (30); it reported no impact on COVID-19 anxiety and lower examination anxiety among an isolated group compared with a non-isolated group. Since the publication of the study, Nigeria has experienced an increase in COVID-19 cases and imposed further societal lockdown measures.

Of the European studies (*n* = 16), half were conducted in Italy (*n* = 8). In February 2020, there was a severe outbreak of COVID-19 in northern Italy, which was placed into lockdown. A national lockdown followed in March 2020, which was gradually eased from May 2020. During the first wave of the pandemic, the number of active cases in Italy was one of the highest in the world. Most of the Italian studies were cross-sectional (*n* = 7) and reported high levels of mental health and well-being issues among children and adolescents during the pandemic, as well as evidence of interaction between parental mental health and child and adolescent mental health (36, 37). The only longitudinal study conducted in Italy reported that, compared with 1 year earlier, adolescents experienced fewer positive emotions and more negative emotions after the COVID-19 national lockdown (54).

Further research from Europe was conducted in Belgium (*n* = 1), Spain (*n* = 4), Germany (*n* = 1), the Netherlands (*n* = 2), and the United Kingdom (*n* = 1). Increased depressive symptoms were reported in the UK (66), there was some evidence of increased psychological stress in Germany (53), the evidence from Spain was mixed (38–40, 55), and only slight impact was interpreted in the Netherlands (56, 57). The Belgian study explored the relationship between anxiety and social media (31). All these European countries have implemented a “lockdown” of some form. Yet, the lockdown approach in the Netherlands has been relatively relaxed compared to other European countries, with the government implementing its so-called “intelligent lockdown” whereby people were asked to stay home but were still allowed to move around freely as long as they kept a distance of 1.5 m to others. The varying “lockdown” approaches could, in part, account for the varying impacts on child and adolescent mental health; yet, it is difficult to conclude this from the available evidence.

There were 13 studies from North America and most of the studies (*n* = 10/13) were conducted in the US and the remaining (*n* = 3/13) in Canada. The majority of the US studies were cross-sectional (*n* = 6/10) and reported: negative association between the pandemic and societal control measures and the mental health of children and adolescents (43, 47); how parental mental health was associated to their children/adolescents' mental health (43, 48) or how parents' and children's well-being in the post-crisis period was strongly associated with the number of crisis-related hardships (such as job loss, income loss, caregiving burden, and illness) that the family experienced (45). One study reported that children's mental health fell within the clinical range, however, mental health symptoms were positively associated with the number of children in the home (44). Oosterhoff et al. (46) did not find any evidence of a potential association between degree of social distancing nor any indicator of mental health. Three of the longitudinal studies reported a significant increase, albeit small, in symptoms of mental ill-health during the pandemic (compared to before the pandemic) (60, 63, 68).

The two Canadian cross-sectional studies reported: 43% of adolescents expressed they were “very concerned” about the pandemic (42) while Carroll et al. (41) reported that, according to parents, almost half (49%) of children had very little concern about COVID-19, 38% were somewhat concerned, and 7% were very much concerned. Browne et al. (59), the only longitudinal study from Canada, reported that male children's mental health problems worsened significantly during the pandemic. No significant differences over time were observed for females.

There were two cross-sectional studies conducted in South America as another geographical region: one from Brazil and the other from Ecuador. Garcia de Avila (49) assessed the prevalence of anxiety among Brazilian school-children and reported a high prevalence of anxiety (19%), especially among children with parents with essential jobs and those who were social distancing without parents. Asanov et al. (50) assessed the mental health of Ecuadorian high-school students during the COVID-19 quarantine and reported that 16% of students had mental health scores that indicated major depression.

The two studies conducted in Australia were both longitudinal studies and reported the negative impact of COVID-19 and associated societal control measures on the mental health of children and adolescents. Magson et al. (64) found significant increases in depression and anxiety symptoms as well as a significant decrease in life satisfaction among adolescents, from before to during the pandemic, which was particularly pronounced among girls. Similarly, Munasinghe et al. (65) investigated changes in well-being during the early period of physical distancing among adolescents and results highlighted that the implementation of physical distancing interventions was associated with decreases in well-being.

### Are There Any Protective Factors Associated With a Lower Likelihood of Mental Health Problem Outcomes?

Some potential protective factors, i.e., characteristics associated with a lower likelihood of negative outcomes or that reduce the negative impact, were identified in the scoping review. With regard to internal protective factors, strong resilience and positive emotion regulation were associated with better mental health outcomes among adolescents (17, 20, 53). On a behavioural level, physical activity was reportedly associated with improved mood among children and adolescents (11, 21). The social environment also appears to play a role, with parental self-efficacy (36), family functioning (62), and emotion regulation (25) as well as level of social support (12) associated with better outcomes.

### Are There Any Factors Associated With a Higher Likelihood of Mental Health Problem Outcomes?

A number of factors associated with poorer mental health outcomes were identified in the scoping review. The level of concern about COVID-19 among adolescents was found to be associated with poorer mental health outcomes (29, 42), and could be related to internal factors such as emotional reactivity and experiential avoidance (29, 53) as well as exposure to excessive information (28). Similarly, greater COVID-19 school concerns were associated with increased depression symptoms (60). The presence of COVID-19 cases in adolescents' communities contributed to poorer mental health, which was more pronounced for older adolescents (13). Several studies indicated that parental mental health problems were related to poorer child and adolescent outcomes (7, 16, 25, 37, 48, 53, 56), which strengthens the evidence that social environment is an important factor. Internet, social media and video game use was another common research topic, evidence from which suggests negative association with child and adolescent mental health (9, 18, 24, 26, 31, 51, 67).

## Discussion

This scoping review brings together all the published studies exploring child and adolescent mental health during the COVID-19 pandemic around the world, published until December 2020, and all longitudinal studies until early May 2021. In just over a year there were 59 studies that met the inclusion criteria for this review. The figure would have been higher if the focus of the second review process had not been narrowed to longitudinal studies only. This highlights the extensive research activity during the pandemic period. Yet, only 15 of the studies adopted a longitudinal research design, which means evidence on how the COVID-19 pandemic and societal control measures have affected the mental health of children and adolescents is still somewhat limited.

The definition of “child” and “adolescent” varied across the included studies; yet, most reportedly involved adolescents (*n* = 30; 63%). Around a third of the studies relied only on parent reports to measure child and adolescent mental health (*n* = 19: 32%). Across all the included studies, a range of outcome measures were used to assess mental health including bespoke questions formulated for the purpose of the research (Tables 2, 3). It is important to highlight here the timing of when data was collected and how it changed from one study to the other: in some of the studies, data were collected at the very beginning of the pandemic (already from February 2020); some other studies collected their data during the first COVID-19 peak (March, April, or May 2020) according to each country; and in some other studies data were collected when the pandemic seemed to be under control and societal control measures were no longer very strict. There is some evidence to suggest that the timing of data collection could affect findings (68).

Most studies reported negative impact of COVID-19 on child and adolescent mental health outcomes, yet the evidence was mixed (Tables 2, 3). This was also the case for studies investigating societal control measures. Strong resilience, positive emotion regulation, physical activity, parental self-efficacy, family functioning and emotional regulation, and social support were reported as protective factors. On the contrary, emotional reactivity and experiential avoidance, exposure to excessive information, COVID-19 school concerns, presence of COVID-19 cases in the community, parental mental health problems, and high internet, social media, and video game use were all identified as potentially harmful factors.

Collating the evidence in a scoping review such as this provides an initial step toward addressing negative impact of the pandemic and child and adolescent mental health. By taking the various findings across the body of research into consideration, interventional strategies can be developed. Taking action now could mitigate longer term impact on the overall health and mental health of children and adolescents. Not only could this be helpful in the present day, but it could also be informative for future pandemics. In terms of the nature of intervention, the aforementioned protective and harmful factors identified in the literature provide grounding for potential intervention targets.

Under this unprecedented and current situation due to the COVID-19 pandemic and societal infection control measures, the levels of physical activity and sedentary behaviour of children and adolescents are important aspects to be considered. Physical activity was shown to be associated with better mood state during the pandemic by two studies (11, 21). Both studies were conducted in China, which somewhat limits the generalisability of the findings. However, the extant literature on physical activity and mental health outcomes is supportive of this relationship: physical activity and mental health (69–73). COVID-19 is an ongoing pandemic that may affect physical activity patterns and sedentary time in the longer-term (74). These results highlight the need for interventions to keep children and adolescents active and fit during the pandemic (74, 75). A number of systematic reviews and meta-analyses assessing the potential of school-based interventions have reported physical activity as a positive and promising strategy to improve child and adolescent mental health (73, 76, 77); however, an alternative approach in the context of a pandemic should be investigated since so many stay-at-home orders are intermittently in place.

Another protective factor identified in the review that could be considered as an intervention target is parental self-efficacy (36). Conversely, parental mental health problems were associated with poorer outcomes among children and adolescents (7, 16, 25, 37, 48). Morelli et al. (36) make the case that, although parents are likely to be exposed to high levels of stress during the pandemic period, support can be offered in how to introduce daily structure and promote positive emotional functioning in their children. There are evidence-based parenting programs that cover these topics (78). Guidance on how to talk to children about the COVID-19 pandemic, including the loss of loved ones, has been published (79) as well as a picture book to read together with children (80).

Spending more time using social media and reading the news had a strong negative association with mental health outcomes (9). Some of the studies discussed that feeling lonely and anxious motivated children and adolescents to use social media more often mainly to cope with the situation and with the lack of social contact; however, it resulted in even more negative feelings of anxiety, depression and loneliness (43). An aspect related to this that should be considered more generally is screen use (81). Excessive screen use is known to impact on sleep and physical activity (82) and has been linked with poorer language skills, lower school performance and classroom engagement, social/emotional difficulties, and reduced psychological well-being (81). Although screen media use among children and adolescents can be used with positive intentions, such as for education or to interact with friends and family and can award parents time to complete necessary tasks, it is an area of concern, especially during the COVID-19 pandemic (81, 83). Research on online social network site (SNS) addiction, such as excessive and compulsive online social networking, is growing (84). Not being a formally recognised diagnosis, well-documented therapeutic interventions are difficult to find. However, interventions and preventive efforts proven effective for other addictive behaviours, such as self-help strategies and therapies, can be applied to SNS addiction (84). In 2019, WHO released guidelines (85) stating that screen time is not recommended for children 1 year or under and for children 2–4 years old, the daily sedentary screen time should not be more than 1 h. Reading and storytelling are promoted as alternative sedentary activities (85).

This review comes with some limitations. Certain requirements that are placed on a more comprehensive systematic review, e.g., that the relevance of all studies must be checked by two independent reviewers, were omitted to be able to compile the findings from the literature in an efficient manner. This entails limitations in both accuracy and the scope of the material. Most of the included studies had a cross-sectional design, and therefore the direction of the association cannot be inferred, as the results do not provide knowledge about causation, but only about mathematical relationships. Yet, the second search performed to update the review with a particular focus on longitudinal studies can be considered a strength. Other methodological limitations of the studies include common use of convenience sampling, parent report in place of direct report in several studies, as well as a lack of validated outcome measures in some studies. Finally, the heterogeneity of the included studies and geographical variation limits comparability, and negates the possibility of meta-analysis. Therefore, only narrative description of the study findings was provided in the review. For all the above limitations, the results should be interpreted with caution. As more studies are added to the body of literature, the formation of the evidence could shift. This overview should therefore be seen as a “snapshot” of the current literature.

## Conclusions

Due to the methodological heterogeneity of the studies included in this scoping review, as well as the low number of longitudinal studies and geographical variation, it is challenging to draw definitive conclusions about the real impact of the COVID-19 pandemic and societal infection control measures on the mental health of children and adolescents. However, the existing body of research gives some insight to potential protective and harmful factors that can be used to inform how parents, clinicians and policy makers can take action to mitigate the effects of the pandemic. From the protective and harmful factors identified in the scoping review, some potential intervention targets have been identified. Namely, interventions to promote physical activity and reduce screen time among children and adolescents, as well as parenting support programs to increase parental self-efficacy and promote positive and warm parent-child relationships.

## Author Contributions

AS conceived the idea for the literature search. JM led on designing the search, with support from GW. JM led on conducting the literature search and analysing the data. GW, NJ, and AS contributed to the interpretation of the data. JM and GW wrote the first draft of the manuscript. NJ and AS critically revised the manuscript for important intellectual content. All authors contributed to and have approved the final manuscript.

## Conflict of Interest

The authors declare that the research was conducted in the absence of any commercial or financial relationships that could be construed as a potential conflict of interest.

## Publisher's Note

All claims expressed in this article are solely those of the authors and do not necessarily represent those of their affiliated organizations, or those of the publisher, the editors and the reviewers. Any product that may be evaluated in this article, or claim that may be made by its manufacturer, is not guaranteed or endorsed by the publisher.

## References

[1] Nadeem MS Zamzami MA Choudhry H Murtaza BN Kazmi I Ahmad H. Origin, potential therapeutic targets and treatment for coronavirus disease (COVID-19). Pathogens. (2020) 9:307. 10.3390/pathogens9040307 32331255 PMC7238035

[2] Hossain MM Sultana A Purohit N. Mental health outcomes of quarantine and isolation for infection prevention: a systematic umbrella review of the global evidence. Epidemiol Health. (2020) 42:e2020038. 10.4178/epih.e2020038 32512661 PMC7644933

[3] Manuell ME Cukor J. Mother Nature versus human nature: public compliance with evacuation and quarantine. Disasters. (2011) 35:417–42. 10.1111/j.1467-7717.2010.01219.x 21073672

[4] Brooks SK Webster RK Smith LE Woodland L Wessely S Greenberg N. The psychological impact of quarantine and how to reduce it: rapid review of the evidence. Lancet. (2020) 395:912–20. 10.1016/S0140-6736(20)30460-8 32112714 PMC7158942

[5] Loades ME Chatburn E Higson-Sweeney N Reynolds S Shafran R Brigden A. Rapid systematic review: the impact of social isolation and loneliness on the mental health of children and adolescents in the context of COVID-19. J Am Acad Child Adolesc Psychiatry. (2020) 59:1218–39.e3. 10.1016/j.jaac.2020.05.009 32504808 PMC7267797

[6] Arskey H O'Malley L. Scoping studies: towards a methodological framework. Int J Soc Res Methodol. (2005) 8:19–32. 10.1080/1364557032000119616

[7] Yeasmin S Banik R Hossain S Hossain MN Mahumud R Salma N. Impact of COVID-19 pandemic on the mental health of children in Bangladesh: a cross-sectional study. Child Youth Serv Rev. (2020) 117:105277. 10.1016/j.childyouth.2020.105277 32834275 PMC7387938

[8] Chen S Cheng Z Wu J. Risk factors for adolescents' mental health during the COVID-19 pandemic: a comparison between Wuhan and other urban areas in China. Global Health. (2020) 16:96. 10.1186/s12992-020-00627-7 33036622 PMC7545801

[9] Dong H Yang F Lu X Hao W. Internet addiction and related psychological factors among children and adolescents in China during the coronavirus disease 2019 (COVID-19) epidemic. Front Psychiatry. (2020) 11:751. 10.3389/fpsyt.2020.00751 32982806 PMC7492537

[10] Duan L Shao X Wang Y Huang Y Miao J Yang X. An investigation of mental health status of children and adolescents in china during the outbreak of COVID-19. J Affect Disord. (2020) 275:112–8. 10.1016/j.jad.2020.06.029 32658812 PMC7329661

[11] Kang S Sun Y Zhang X Sun F Wang B Zhu W. Is physical activity associated with mental health among Chinese adolescents during isolation in COVID-19 pandemic? J Epidemiol Glob Health. (2020) 11:26–33. 32959611 10.2991/jegh.k.200908.001 PMC7958283

[12] Qi MMS Zhou S-JMS Guo Z-C Zhang L-G Min H-J Li X-MMS. The effect of social support on mental health in Chinese adolescents during the outbreak of COVID-19. J Adolescent Health. (2020) 67:514. 10.1016/j.jadohealth.2020.07.001 32753347 PMC7395830

[13] Ren H He X Bian X Shang X Liu J. The protective roles of exercise and maintenance of daily living routines for Chinese adolescents during the COVID-19 quarantine period. J Adolesc Health. (2020) 68:35–42. 33121902 10.1016/j.jadohealth.2020.09.026

[14] Shuang-Jiang Z Li-Gang Z Lei-Lei W Zhao-Chang G Jing-Qi W Jin-Cheng C. Prevalence and socio-demographic correlates of psychological health problems in Chinese adolescents during the outbreak of COVID-19. Euro Child Adolescent Psychiatry. (2020) 29:749–58. 10.1007/s00787-020-01541-4 32363492 PMC7196181

[15] Tang S Xiang M Cheung T Xiang YT. Mental health and its correlates among children and adolescents during COVID-19 school closure: the importance of parent-child discussion. J Affect Disord. (2020) 279:353–60. 10.1016/j.jad.2020.10.016 33099049 PMC7550131

[16] Tso WWY Wong RS Tung KTS Rao N Fu KW Yam JCS. Vulnerability and resilience in children during the COVID-19 pandemic. Eur Child Adolesc Psychiatry. (2020). [Epub ahead of print]. 10.1007/s00787-020-01680-8 PMC7671186 33205284

[17] Yang D Swekwi U Tu CC Dai X. Psychological effects of the COVID-19 pandemic on Wuhan's high school students. Child Youth Serv Rev. (2020) 119:105634. 10.1016/j.childyouth.2020.105634 33162628 PMC7603991

[18] Yue J Zang X Le Y An Y. Anxiety, depression and PTSD among children and their parent during 2019 novel coronavirus disease (COVID-19) outbreak in China. Curr Psychol. (2020). [Epub ahead of print]. 10.1007/s12144-020-01191-4 PMC7666617 33223783

[19] Zhang C Ye M Fu Y Yang M Luo F Yuan J. The psychological impact of the COVID-19 pandemic on teenagers in China. J Adolesc Health. (2020) 67:747–55. 10.1016/j.jadohealth.2020.08.026 33041204 PMC7543885

[20] Zhang Q Zhou L Xia J. Impact of COVID-19 on emotional resilience and learning management of middle school students. Med Sci Monit. (2020) 26:e924994. 10.12659/MSM.924994 32869770 PMC7485285

[21] Zhang X Zhu W Kang S Qiu L Lu Z Sun Y. Association between physical activity and mood states of children and adolescents in social isolation during the COVID-19 epidemic. Int J Environ Res Public Health. (2020) 17:1–12. 10.3390/ijerph17207666 33096659 PMC7589310

[22] Zhou J Yuan X Qi H Liu R Li Y Huang H. Prevalence of depression and its correlative factors among female adolescents in China during the coronavirus disease 2019 outbreak. Global Health. (2020) 16:69. 10.1186/s12992-020-00601-3 32723373 PMC7385712

[23] Darvishi E Golestan S Demehri F Jamalnia S. A cross-sectional study on cognitive errors and obsessive-compulsive disorders among young people during the outbreak of coronavirus disease 2019. Act Nerv Super. (2007) 2020:1–6. 10.1007/s41470-020-00077-x 33163111 PMC7602764

[24] Fazeli S Mohammadi Zeidi I Lin CY Namdar P Griffiths MD Ahorsu DK. Depression, anxiety, and stress mediate the associations between internet gaming disorder, insomnia, and quality of life during the COVID-19 outbreak. Addict Behav Rep. (2020) 12:100307. 10.1016/j.abrep.2020.100307 33110934 PMC7581367

[25] Shorer M Leibovich L. Young children's emotional stress reactions during the COVID-19 outbreak and their associations with parental emotion regulation and parental playfulness. Early Child Dev Care. (2020) 11:1–11. 10.1080/03004430.2020.1806830

[26] Lin MP. Prevalence of internet addiction during the COVID-19 outbreak and its risk factors among junior high school students in Taiwan. Int J Environ Res Public Health. (2020) 17:1–12. 10.3390/ijerph17228547 33218018 PMC7698622

[27] Adibelli D Sümen A. The effect of the coronavirus (COVID-19) pandemic on health-related quality of life in children. Child Youth Serv Rev. (2020) 119:105595. 10.1016/j.childyouth.2020.105595 33071408 PMC7550976

[28] Kilinçel S Kilinçel O Muratdagi G Aydin A Usta MB. Factors affecting the anxiety levels of adolescents in home-quarantine during COVID-19 pandemic in Turkey. Asia Pac Psychiatry. (2020). [Epub ahead of print]. 10.1111/appy.12406 PMC7435562 32783389

[29] Seçer I Ulaş S. An investigation of the effect of COVID-19 on OCD in youth in the context of emotional reactivity, experiential avoidance, depression and anxiety. Int J Ment Health Addict. (2020) 1–14. 10.1007/s11469-020-00322-z 32837429 PMC7293436

[30] Rakhmanov O Shaimerdenov Y Dane S. The effects of COVID-19 pandemic on anxiety in secondary school students. J Res Med Dent Sci. (2020) 8:186–90. Available online at: https://www.jrmds.in/articles/the-effects-of-covid19-pandemic-on-anxiety-in-secondary-school-students-58209.html

[31] Cauberghe V Van Wesenbeeck I De Jans S Hudders L Ponnet K. How adolescents use social media to cope with feelings of loneliness and anxiety during COVID-19 lockdown. Cyberpsychol Behav Soc Netw. (2020) 24:250–7. 33185488 10.1089/cyber.2020.0478

[32] Crescentini C Feruglio S Matiz A Paschetto A Vidal E Cogo P. Stuck outside and inside: an exploratory study on the effects of the COVID-19 outbreak on Italian parents and children's internalizing symptoms. Front Psychol. (2020) 11:586074. 10.3389/fpsyg.2020.586074 33192917 PMC7649806

[33] Cusinato M Iannattone S Spoto A Poli M Moretti C Gatta M. Stress, resilience, and well-being in italian children and their parents during the COVID-19 pandemic. Int J Environ Res Public Health. (2020) 17:1–17. 10.3390/ijerph17228297 33182661 PMC7696524

[34] di Cagno A Buonsenso A Baralla F Grazioli E Di Martino G Lecce E. Psychological impact of the quarantine-induced stress during the coronavirus (COVID-19) outbreak among Italian athletes. Int J Environ Res Public Health. (2020) 17:1–13. 10.3390/ijerph17238867 33260584 PMC7730741

[35] Di Giorgio E Di Riso D Mioni G Cellini N. The interplay between mothers' and children behavioral and psychological factors during COVID-19: an Italian study. Eur Child Adolesc Psychiatry. (2020). [Epub ahead of print]. 10.1007/s00787-020-01631-3 PMC7456665 32865654

[36] Morelli M Cattelino E Baiocco R Trumello C Babore A Candelori C. Parents and children during the COVID-19 lockdown: the influence of parenting distress and parenting self-efficacy on children's emotional well-being. Front Psychol. (2020) 11:584645. 10.3389/fpsyg.2020.584645 33123063 PMC7574609

[37] Spinelli M Lionetti F Pastore M Fasolo M. Parents' stress and children's psychological problems in families facing the COVID-19 outbreak in Italy. Front Psychol. (2020) 11:1713. 10.3389/fpsyg.2020.01713 32719646 PMC7350926

[38] Orgilés M Morales A Delvecchio E Mazzeschi C Espada JP. Immediate psychological effects of the COVID-19 quarantine in youth from Italy and Spain. Front Psychol. (2020) 11:579038. 10.3389/fpsyg.2020.579038 33240167 PMC7677301

[39] Ezpeleta L Navarro JB de la Osa N Trepat E Penelo E. Life Conditions during COVID-19 lockdown and mental health in Spanish adolescents. Int J Environ Res Public Health. (2020) 17:1–11. 10.3390/ijerph17197327 33036461 PMC7579639

[40] Romero E López-Romero L Domínguez-Álvarez B Villar P Gómez-Fraguela JA. Testing the effects of COVID-19 confinement in spanish children: the role of parents' distress, emotional problems and specific parenting. Int J Environ Res Public Health. (2020) 17:1–23. 10.3390/ijerph17196975 32987641 PMC7578923

[41] Carroll N Sadowski A Laila A Hruska V Nixon M Ma DWL. The impact of COVID-19 on health behavior, stress, financial and food security among middle to high income Canadian families with young children. Nutrients. (2020) 12:1–14. 10.3390/nu12082352 32784530 PMC7468859

[42] Ellis WE Dumas TM Forbes LM. Physically isolated but socially connected: psychological adjustment and stress among adolescents during the initial COVID-19 crisis. Can J Behav Sci Rev Can Sci Comport. (2020) 52:177–87. 10.1037/cbs0000215

[43] Drouin M McDaniel BT Pater J Toscos T. How parents and their children used social media and technology at the beginning of the COVID-19 pandemic and associations with anxiety. Cyberpsychol Behav Soc Netw. (2020) 23:727–36. 10.1089/cyber.2020.0284 32726144

[44] Fitzpatrick O Carson A Weisz JR. Using mixed methods to identify the primary mental health problems and needs of children, adolescents, and their caregivers during the coronavirus (COVID-19) pandemic. Child Psychiatry Hum Dev. (2020). [Epub ahead of print]. 10.1007/s10578-020-01089-z PMC7590914 33108612

[45] Gassman-Pines A Ananat EO Fitz-Henley J 2nd. COVID-19 and parent-child psychological well-being. Pediatrics. (2020) 146:1–9. 10.1542/peds.2020-007294 32764151 PMC7546085

[46] Oosterhoff BP Palmer CAP Wilson JMS Shook NP. Adolescents' motivations to engage in social distancing during the COVID-19 pandemic: associations with mental and social health. J Adolescent Health. (2020) 67:179. 10.1016/j.jadohealth.2020.05.004 32487491 PMC7205689

[47] Patrick SW Henkhaus LE Zickafoose JS Lovell K Halvorson A Loch S. Well-being of parents and children during the COVID-19 pandemic: a national survey. Pediatrics. (2020) 146:1–8. 10.1542/peds.2020-016824 32709738

[48] Russell BS Hutchison M Tambling R Tomkunas AJ Horton AL. Initial challenges of caregiving during COVID-19: caregiver burden, mental health, and the parent-child relationship. Child Psychiatry Hum Dev. (2020) 51:671–82. 10.1007/s10578-020-01037-x 32749568 PMC7398861

[49] Garcia de Avila MA Hamamoto Filho PT Jacob F Alcantara LRS Berghammer M Jenholt Nolbris M. Children's anxiety and factors related to the COVID-19 pandemic: an exploratory study using the children's anxiety questionnaire and the numerical rating scale. Int J Environ Res Public Health. (2020) 17:1–13. 10.3390/ijerph17165757 32784898 PMC7459447

[50] Asanov I Flores F McKenzie D Mensmann M Schulte M. Remote-learning, time-use, and mental health of Ecuadorian high-school students during the COVID-19 quarantine. World Dev. (2021) 138:105225. 10.1016/j.worlddev.2020.105225 33110286 PMC7581322

[51] Chen I-H Chen C-Y Pakpour AH Griffiths MD Lin C-Y Li X-D. Problematic internet-related behaviors mediate the associations between levels of internet engagement and distress among schoolchildren during COVID-19 lockdown: a longitudinal structural equation modeling study. J Behav Addictions JBA. (2021) 10:135–48. 10.1556/2006.2021.00006 33570506 PMC8969851

[52] Teng Z Pontes HM Nie Q Griffiths MD Guo C. Depression and anxiety symptoms associated with internet gaming disorder before and during the COVID-19 pandemic: a longitudinal study. J Behav Addict. (2021) 10:169–80. 33704085 10.1556/2006.2021.00016 PMC8969853

[53] Paschke K Arnaud N Austermann MI Thomasius R. Risk factors for prospective increase in psychological stress during COVID-19 lockdown in a representative sample of adolescents and their parents. BJPsych Open. (2021) 7:e94. 10.1192/bjo.2021.49 33938424 PMC8111205

[54] Alivernini F Manganelli S Girelli L Cozzolino M Lucidi F Cavicchiolo E. Physical distancing behavior: the role of emotions, personality, motivations, and moral decision-making. J Pediatric Psychol. (2020) 46:15–26. 10.1093/jpepsy/jsaa122 33355343 PMC7798981

[55] Giménez-Dasí M Quintanilla L Lucas-Molina B Sarmento-Henrique R. Six weeks of confinement: psychological effects on a sample of children in early childhood and primary education. Front Psychol. (2020) 11:590463. 10.3389/fpsyg.2020.590463 33132994 PMC7578387

[56] Achterberg M Dobbelaar S Boer OD Crone EA. Perceived stress as mediator for longitudinal effects of the COVID-19 lockdown on wellbeing of parents and children. Sci Rep. (2021) 11:2971. 10.1038/s41598-021-81720-8 33536464 PMC7859207

[57] Janssen LHC Kullberg MJ Verkuil B van Zwieten N Wever MCM van Houtum L. Does the COVID-19 pandemic impact parents' and adolescents' well-being? An EMA-study on daily affect and parenting. PLoS ONE. (2020) 15:e0240962. 10.1371/journal.pone.0240962 33064778 PMC7567366

[58] Bignardi G Dalmaijer ES Anwyl-Irvine AL Smith TA Siugzdaite R Uh S. Longitudinal increases in childhood depression symptoms during the COVID-19 lockdown. Arch Dis Childhood. (2020) 106:791–7. 33298552 10.1136/archdischild-2020-320372 PMC7733224

[59] Browne DT Wade M May SS Maguire N Wise D Estey K. Children's mental health problems during the initial emergence of COVID-19. Can Psychol. (2021) 62:65–72. 10.1037/cap0000273

[60] Hawes MT Szenczy AK Klein DN Hajcak G Nelson BD. Increases in depression and anxiety symptoms in adolescents and young adults during the COVID-19 pandemic. Psychol Med. (2021). [Epub ahead of print]. 10.1017/S0033291720005358 PMC7844180 33436120

[61] Hawes MT Szenczy AK Olino TM Nelson BD Klein DN. Trajectories of depression, anxiety and pandemic experiences; a longitudinal study of youth in New York during the Spring-Summer of 2020. Psychiatry Res. (2021) 298:113778. 10.1016/j.psychres.2021.113778 33550176 PMC9754702

[62] Penner F Hernandez Ortiz J Sharp C. Change in youth mental health during the COVID-19 pandemic in a majority hispanic/latinx US sample. J Am Acad Child Adolesc Psychiatry. (2021) 60:513–23. 10.1016/j.jaac.2020.12.027 33359408

[63] Rogers AA Ha T Ockey S. Adolescents' perceived socio-emotional impact of COVID-19 and implications for mental health: results from a U.S.-based mixed-methods study. J Adolesc Health. (2020) 68:43–52. 33143986 10.1016/j.jadohealth.2020.09.039 PMC7605752

[64] Magson NR Freeman JYA Rapee RM Richardson CE Oar EL Fardouly J. Risk and protective factors for prospective changes in adolescent mental health during the COVID-19 pandemic. J Youth Adolesc. (2020) 50:1–14. 10.1007/s10964-020-01332-9 33108542 PMC7590912

[65] Munasinghe S Sperandei SP Freebairn LP Conroy EP Jani HM Marjanovic SM. The impact of physical distancing policies during the COVID-19 pandemic on health and well-being among australian adolescents. J Adolescent Health. (2020) 67:653. 10.1016/j.jadohealth.2020.08.008 33099413 PMC7577185

[66] Bignardi G Dalmaijer ES Anwyl-Irvine AL Smith TA Siugzdaite R Uh S. Longitudinal increases in childhood depression symptoms during the COVID-19 lockdown. Arch Dis Childhood. (2020). 33298552 10.1136/archdischild-2020-320372 PMC7733224

[67] Teng Z Pontes HM Nie Q Griffiths MD Guo C. Depression and anxiety symptoms associated with internet gaming disorder before and during the COVID-19 pandemic: a longitudinal study. J Behav Addict. (2021). 33704085 10.1556/2006.2021.00016 PMC8969853

[68] Hawes MT Szenczy AK Olino TM Nelson BD Klein DN. Trajectories of depression, anxiety and pandemic experiences; a longitudinal study of youth in New York during the Spring-Summer of 2020. Psychiatry Res. (2021) 298:113778. 33550176 10.1016/j.psychres.2021.113778 PMC9754702

[69] Rodriguez-Ayllon M Cadenas-Sánchez C Estévez-López F Muñoz NE Mora-Gonzalez J Migueles JH. Role of physical activity and sedentary behavior in the mental health of preschoolers, children and adolescents: a systematic review and meta-analysis. Sports Med. (2019) 49:1383–410. 10.1007/s40279-019-01099-5 30993594

[70] Radovic S Gordon MS Melvin GA. Should we recommend exercise to adolescents with depressive symptoms? A meta-analysis. J Paediatrics Child Health. (2017) 53:214–20. 10.1111/jpc.13426 28070942

[71] Dale LP Vanderloo L Moore S Faulkner G. Physical activity and depression, anxiety, and self-esteem in children and youth: an umbrella systematic review. Mental Health Phys Activity. (2019) 16:66–79. 10.1016/j.mhpa.2018.12.001

[72] Korczak DJ Madigan S Colasanto M. Children's physical activity and depression: a meta-analysis. Pediatrics. (2017) 139:1–14. 10.1542/peds.2016-2266 28314824

[73] Wegner M Amatriain-Fernández S Kaulitzky A Murillo-Rodriguez E Machado S Budde H. Systematic review of meta-analyses: exercise effects on depression in children and adolescents. Front Psychiatry. (2020) 11:81. 10.3389/fpsyt.2020.00081 32210847 PMC7068196

[74] Xiang M Zhang Z Kuwahara K. Impact of COVID-19 pandemic on children and adolescents' lifestyle behavior larger than expected. Prog Cardiovasc Dis. (2020) 63:531–2. 10.1016/j.pcad.2020.04.013 32360513 PMC7190470

[75] Pavlovic A DeFina LF Natale BL Thiele SE Walker TJ Craig DW. Keeping children healthy during and after COVID-19 pandemic: meeting youth physical activity needs. BMC Public Health. (2021) 21:485. 10.1186/s12889-021-10545-x 33706744 PMC7948663

[76] Andermo S Hallgren M Nguyen T-T-D Jonsson S Petersen S Friberg M. School-related physical activity interventions and mental health among children: a systematic review and meta-analysis. Sports Med Open. (2020) 6:25. 10.1186/s40798-020-00254-x 32548792 PMC7297899

[77] Brown HE Pearson N Braithwaite RE Brown WJ Biddle SJ. Physical activity interventions and depression in children and adolescents: a systematic review and meta-analysis. Sports Med. (2013) 43:195–206. 10.1007/s40279-012-0015-8 23329611

[78] Barlow J Coren E. The effectiveness of parenting programs: a review of campbell reviews. Res Social Work Prac. (2018) 28:99–102. 10.1177/1049731517725184

[79] Rapa E Dalton L Stein A. Talking to children about illness and death of a loved one during the COVID-19 pandemic. Lancet Child Adolescent Health. (2020) 560–2. 10.1016/S2352-4642(20)30174-7 32505223 PMC7272150

[80] Wilson K Jenner E Roberts N. Coronavirus: A Book for Children: Nosy Crow. London: Nosy Crow Ltd (2020).

[81] Stienwandt S Cameron EE Soderstrom M Casar MJ Le C Roos LE. Keeping kids busy: family factors associated with hands-on play and screen time during the COVID-19 pandemic. PsyArXiv. (2020) 1–45. 10.31234/osf.io/prtyf PMC8731198 35013660

[82] Arufe-Giráldez V Sanmiguel-Rodríguez A Zagalaz-Sánchez ML Cachón-Zagalaz J González-Valero G. Sleep, physical activity and screens in 0-4 years Spanish children during the COVID-19 pandemic: were the WHO recommendations met? J Hum Sport Exerc. (2020). 10.14198/jhse.2022.173.02

[83] Lau EYH Lee K. Parents' views on young children's distance learning and screen time during COVID-19 class suspension in Hong Kong. Early Educ Dev. (2020) 32:6. 10.1080/10409289.2020.1843925

[84] Andreassen CS. Online social network site addiction: a comprehensive review. Curr Addict Rep. (2015) 2:175–84. 10.1007/s40429-015-0056-9

[85] World Health O. Guidelines on Physical Activity, Sedentary Behaviour and Sleep for Children Under 5 Years of Age. Geneva: World Health Organization (2019). 31091057

