# Supplementary material for: The Impact of the COVID-19 Pandemic and Societal Infection Control Measures on Children and Adolescents' Mental Health: A Scoping Review
Source: Front Psychiatry. 2021 Sep 6;12:711791. doi: 10.3389/fpsyt.2021.711791 (PMC8451953; doi:10.3389/fpsyt.2021.711791)
Supplement: Supplementary file 1 [file Table_1.pdf]

APA PsycInfo 2020-12-04

Database: APA PsycInfo, EBSCO

Date: 2020-12-04

Number	Search terms	Results	Comment
1	TI (Adolesc* OR Preadolesc* OR Pre-adolesc* OR Boy OR Boys OR Boyhood OR Girl* OR Child OR Children OR Childhood OR Kid OR Kids OR Toddler* OR Preschool* OR Pre-school* OR Teen* OR Young* OR Youth* OR School* OR "Junior high*" OR "High-school*" OR Highschool* OR "Senior high" OR Schoolchild* OR Kindergarten* OR Playgroup* OR Play-group* OR Playschool*) OR AB (Adolesc* OR Preadolesc* OR Pre-adolesc* OR Boy OR Boys OR Boyhood OR Girl* OR Child OR Children OR Childhood OR Kid OR Kids OR Toddler* OR Preschool* OR Pre-school* OR Teen* OR Young* OR Youth* OR School* OR "Junior high*" OR "High-school*" OR Highschool* OR "Senior high" OR Schoolchild* OR Kindergarten* OR Playgroup* OR Play-group* OR Playschool*)	1,232,974	
2	DE "Coronavirus" OR DE "Pandemics"	2,076	
3	TI (Corona* OR cov2 OR COVID19 OR COVID-19 OR nCov OR Pandemic* OR SARS-CoV-2 OR 2019-nCoV*) OR AB (Corona* OR cov2 OR COVID19 OR COVID-19 OR nCov OR Pandemic* OR SARS-CoV-2 OR 2019-nCoV*)	16,523	
4	S2 OR S3	16,586	
5	DE "Mental Disorders" OR DE "Moodiness" OR DE "Depression (Emotion)" OR DE "Mental Health" OR DE "Behavior Disorders" OR DE "Anxiety" OR DE "Anxiety Disorders" OR DE "Nervousness" OR DE "Stress" OR DE "Anger" OR DE "Suicide"	404,306	
6	TI ("Mental Disorder*" OR "Mental Disease*" OR "Mental Illness*" OR "Mental Instabilit*" OR "Psychiatric Disorder*" OR "Psychiatric Disease*" OR "Psychiatric Illness*" OR "Psychiatric Diagnosis" OR "Mood disorder*" OR "Affective Disorder*" OR "Behavior Disorder*" OR Depressi* OR "Mental Health" OR "Well Being" OR "Well-Being" OR Anxiet* OR Nervous* OR Worr* OR Stress* OR Distress* OR Anger* OR Angry OR Suicide*) OR AB ("Mental Disorder*" OR "Mental Disease*" OR "Mental Illness*" OR "Mental Instabilit*" OR "Psychiatric Disorder*" OR "Psychiatric Disease*" OR "Psychiatric Illness*" OR "Psychiatric Diagnosis" OR "Mood disorder*" OR "Affective Disorder*" OR "Behavior Disorder*" OR Depressi* OR "Mental Health" OR "Well Being" OR "Well-Being" OR Anxiet* OR Nervous* OR Worr* OR Stress* OR Distress* OR Anger* OR Angry OR Suicide*)	996,051	
7	S5 OR S6	1,062,579	
8	S1 AND S4 AND S7	896	
9	S8 NOT Coronary	441	
10	S9 AND Publication Date: 20191201 - 20201231	318	

CINAHL 2020-12-04

Database: CINAHL, EBSCO

Date: 2020-12-04

Number	Search terms	Results	Comment
1	MH "Child, Preschool" OR MH "Child" OR MH "Adolescence"	843,674	
2	TI (Adolesc* OR Preadolesc* OR Pre-adolesc* OR Boy OR Boys OR Boyhood OR Girl* OR Child OR Children OR Childhood OR Kid OR Kids OR Toddler* OR Preschool* OR Pre-school* OR Teen* OR Young* OR Youth* OR School* OR "Junior high*" OR "High-school*" OR Highschool* OR "Senior high" OR Schoolchild* OR Kindergarten* OR Playgroup* OR Play-group* OR Playschool*) OR AB (Adolesc* OR Preadolesc* OR Pre-adolesc* OR Boy OR Boys OR Boyhood OR Girl* OR Child OR Children OR Childhood OR Kid OR Kids OR Toddler* OR Preschool* OR Pre-school* OR Teen* OR Young* OR Youth* OR School* OR "Junior high*" OR "High-school*" OR Highschool* OR "Senior high" OR Schoolchild* OR Kindergarten* OR Playgroup* OR Play-group* OR Playschool*)	809,708	
3	S1 OR S2	1,185,412	
4	MH "Coronavirus Infections" OR MH "COVID-19" OR MH "Disease Outbreaks"	47,741	
5	TI (Corona* OR cov2 OR COVID19 OR COVID-19 OR nCov OR Pandemic* OR SARS-CoV-2 OR 2019-nCoV*) OR AB (Corona* OR cov2 OR COVID19 OR COVID-19 OR nCov OR Pandemic* OR SARS-CoV-2 OR 2019-nCoV*)	136,128	
6	S4 OR S5	162,464	
7	MH "Mental Disorders" OR MH "Affective Disorders" OR MH "Depression" OR MH "Mental Health" OR MH "Anxiety" OR MH "Anxiety Disorders" OR MH "Anger" OR MH "Suicide"	242,286	
8	TI ("Mental Disorder*" OR "Mental Disease*" OR "Mental Illness*" OR "Mental Instabilit*" OR "Psychiatric Disorder*" OR "Psychiatric Disease*" OR "Psychiatric Illness*" OR "Psychiatric Diagnosis" OR "Mood disorder*" OR "Affective Disorder*" OR "Behavior Disorder*" OR Depressi* OR "Mental Health" OR "Well Being" OR "Well-Being" OR Anxiet* OR Nervous* OR Worr* OR Stress* OR Distress* OR Anger* OR Angry OR Suicide*) OR AB ("Mental Disorder*" OR "Mental Disease*" OR "Mental Illness*" OR "Mental Instabilit*" OR "Psychiatric Disorder*" OR "Psychiatric Disease*" OR "Psychiatric Illness*" OR "Psychiatric Diagnosis" OR "Mood disorder*" OR "Affective Disorder*" OR "Behavior Disorder*" OR Depressi* OR "Mental Health" OR "Well Being" OR "Well-Being" OR Anxiet* OR Nervous* OR Worr* OR Stress* OR Distress* OR Anger* OR Angry OR Suicide*)	542,808	
9	S7 OR S8	610,496	
10	S3 AND S6 AND S9	1,700	
11	S10 NOT Coronary	880	
12	S11 AND Publication Date: 20191201 - 20201231	542	

PubMed 2020-12-04

Database: PubMed, National Library of Medicine

Date: 2020-12-04

Number	Search terms	Results	Comment
1	Child, Preschool[MeSH Terms] OR Child[MeSH Terms] OR Adolescent[MeSH Terms]	3,054,252	
2	Adolesc*[Title/Abstract] OR Preadolesc*[Title/Abstract] OR Pre-adolesc*[Title/Abstract] OR Boy[Title/Abstract] OR Boys[Title/Abstract] OR Boyhood[Title/Abstract] OR Girl*[Title/Abstract] OR Child[Title/Abstract] OR Children[Title/Abstract] OR Childhood[Title/Abstract] OR Kid[Title/Abstract] OR Kids[Title/Abstract] OR Toddler*[Title/Abstract] OR Preschool*[Title/Abstract] OR Pre-school*[Title/Abstract] OR Teen*[Title/Abstract] OR Young*[Title/Abstract] OR Youth*[Title/Abstract] OR School*[Title/Abstract] OR "Junior high*"[Title/Abstract] OR "High-school*"[Title/Abstract] OR Highschool*[Title/Abstract] OR "Senior high"[Title/Abstract] OR Schoolchild*[Title/Abstract] OR Kindergarten*[Title/Abstract] OR Playgroup*[Title/Abstract] OR Play-group*[Title/Abstract] OR Playschool*[Title/Abstract]	2,373,330	
3	1 OR 2	4,073,752	
4	Coronavirus Infection[MeSH Terms] OR Pandemics[MeSH Terms]	54,371	
5	Corona*[Title/Abstract] OR cov2[Title/Abstract] OR COVID19[Title/Abstract] OR COVID-19[Title/Abstract] OR nCov[Title/Abstract] OR Pandemic*[Title/Abstract] OR SARS-CoV-2[Title/Abstract] OR 2019-nCoV*[Title/Abstract]	561,666	
6	4 OR 5	568,370	
7	Mental Disorders[MeSH Terms] OR Mood Disorders[MeSH Terms] OR Depression[MeSH Terms] OR Depressive Disorder[MeSH Terms] OR Mental Health[MeSH Terms] OR Anxiety[MeSH Terms] OR Anxiety Disorders[MeSH Terms] OR Anger[MeSH Terms] OR Suicide[MeSH Terms]	1,438,775	
8	"Mental Disorder*"[Title/Abstract] OR "Mental Disease*"[Title/Abstract] OR "Mental Illness*"[Title/Abstract] OR "Mental Instabilit*"[Title/Abstract] OR "Psychiatric Disorder*"[Title/Abstract] OR "Psychiatric Disease*"[Title/Abstract] OR "Psychiatric Illness*"[Title/Abstract] OR "Psychiatric Diagnosis"[Title/Abstract] OR "Mood disorder*"[Title/Abstract] OR "Affective Disorder*"[Title/Abstract] OR "Behavior Disorder*"[Title/Abstract] OR Depressi*[Title/Abstract] OR "Mental Health"[Title/Abstract] OR "Well Being"[Title/Abstract] OR "Well-Being"[Title/Abstract] OR Anxiet*[Title/Abstract] OR Nervous*[Title/Abstract] OR Worr*[Title/Abstract] OR Stress*[Title/Abstract] OR Distress*[Title/Abstract] OR Anger*[Title/Abstract] OR Angry[Title/Abstract] OR Suicide*[Title/Abstract]	1,996,459	
9	7 OR 8	2,910,074	
10	3 AND 6 AND 9	6,126	
11	10 NOT Coronary[Title/Abstract]	2,705	
12	11 AND ("2019/12/01"[Date - Publication] : "3000"[Date - Publication])	1,586	
13	(((((Child, Preschool[MeSH Terms] OR Child[MeSH Terms] OR Adolescent[MeSH Terms]) OR (Adolesc*[Title/Abstract] OR Preadolesc*[Title/Abstract] OR Pre-adolesc*[Title/Abstract] OR Boy[Title/Abstract] OR Boys[Title/Abstract] OR Boyhood[Title/Abstract] OR Girl*[Title/Abstract] OR Child[Title/Abstract] OR Children[Title/Abstract] OR Childhood[Title/Abstract] OR Kid[Title/Abstract] OR Kids[Title/Abstract] OR Toddler*[Title/Abstract] OR Preschool*[Title/Abstract] OR Pre-school*[Title/Abstract] OR Teen*[Title/Abstract] OR Young*[Title/Abstract] OR Youth*[Title/Abstract] OR School*[Title/Abstract] OR "Junior high*"[Title/Abstract] OR "High-school*"[Title/Abstract] OR Highschool*[Title/Abstract] OR "Senior high"[Title/Abstract] OR Schoolchild*[Title/Abstract] OR Kindergarten*[Title/Abstract] OR Playgroup*[Title/Abstract] OR Play-group*[Title/Abstract] OR Playschool*[Title/Abstract])) AND ((Coronavirus Infection[MeSH Terms] OR Pandemics[MeSH Terms]) OR (Corona*[Title/Abstract] OR cov2[Title/Abstract] OR COVID19[Title/Abstract] OR COVID-19[Title/Abstract] OR nCov[Title/Abstract] OR Pandemic*[Title/Abstract] OR SARS-CoV-2[Title/Abstract] OR 2019-nCoV*[Title/Abstract]))) AND ((Mental Disorders[MeSH Terms] OR Mood Disorders[MeSH Terms] OR Depression[MeSH Terms] OR Depressive Disorder[MeSH Terms] OR Mental Health[MeSH Terms] OR Anxiety[MeSH Terms] OR Anxiety Disorders[MeSH Terms] OR Anger[MeSH Terms] OR Suicide[MeSH Terms]) OR ("Mental Disorder*"[Title/Abstract] OR "Mental Disease*"[Title/Abstract] OR "Mental Illness*"[Title/Abstract] OR "Mental Instabilit*"[Title/Abstract] OR "Psychiatric Disorder*"[Title/Abstract] OR "Psychiatric Disease*"[Title/Abstract] OR "Psychiatric Illness*"[Title/Abstract] OR "Psychiatric Diagnosis"[Title/Abstract] OR "Mood disorder*"[Title/Abstract] OR "Affective Disorder*"[Title/Abstract] OR "Behavior Disorder*"[Title/Abstract] OR Depressi*[Title/Abstract] OR "Mental Health"[Title/Abstract] OR "Well Being"[Title/Abstract] OR "Well-Being"[Title/Abstract] OR Anxiet*[Title/Abstract] OR Nervous*[Title/Abstract] OR Worr*[Title/Abstract] OR Stress*[Title/Abstract] OR Distress*[Title/Abstract] OR Anger*[Title/Abstract] OR Angry[Title/Abstract] OR Suicide*[Title/Abstract]))) NOT (Coronary[Title/Abstract])) AND (("2019/12/01"[Date - Publication] : "3000"[Date - Publication]))		Search string

Social Science Premium Collection 2020-12-04

Database: Social Science Premium Collection, ProQuest

Date: 2020-12-04

Number	Search terms	Results	Comment
1	MAINSUBJECT.EXACT("Preschool Children") OR MAINSUBJECT.EXACT("Children") OR MAINSUBJECT.EXACT ("Adolescents")	387,048	
2	Adolesc* OR Preadolesc* OR Pre-adolesc* OR Boy OR Boys OR Boyhood OR Girl* OR Child OR Children OR Childhood OR Kid OR Kids OR Toddler* OR Preschool* OR Pre-school* OR Teen* OR Young* OR Youth* OR School* OR "Junior high*" OR "High-school*" OR Highschool* OR "Senior high" OR Schoolchild* OR Kindergarten* OR Playgroup* OR Play-group* OR Playschool*	3,355,630	
3	1 OR 2	3,370,734	
4	ti(Corona* OR cov2 OR COVID19 OR COVID-19 OR nCov OR Pandemic* OR SARS-CoV-2 OR 2019-nCoV*) OR ab(Corona* OR cov2 OR COVID19 OR COVID-19 OR nCov OR Pandemic* OR SARS-CoV-2 OR 2019-nCoV*)	100,275	
5	MAINSUBJECT.EXACT("Depression (Psychology)") OR MAINSUBJECT.EXACT("Mental Health") OR MAINSUBJECT.EXACT ("Anxiety") OR MAINSUBJECT.EXACT("Anger") OR MAINSUBJECT.EXACT("Suicide")	192,714	
6	ti("Mental Disorder*" OR "Mental Disease*" OR "Mental Illness*" OR "Mental Instabilit*" OR "Psychiatric Disorder*" OR "Psychiatric Disease*" OR "Psychiatric Illness*" OR "Psychiatric Diagnosis" OR "Mood disorder*" OR "Affective Disorder*" OR "Behavior Disorder*" OR Depressi* OR "Mental Health" OR "Well Being" OR "Well-Being" OR Anxiet* OR Nervous* OR Worr* OR Stress* OR Distress* OR Anger* OR Angry OR Suicide*) OR ab("Mental Disorder*" OR "Mental Disease*" OR "Mental Illness*" OR "Mental Instabilit*" OR "Psychiatric Disorder*" OR "Psychiatric Disease*" OR "Psychiatric Illness*" OR "Psychiatric Diagnosis" OR "Mood disorder*" OR "Affective Disorder*" OR "Behavior Disorder*" OR Depressi* OR "Mental Health" OR "Well Being" OR "Well-Being" OR Anxiet* OR Nervous* OR Worr* OR Stress* OR Distress* OR Anger* OR Angry OR Suicide*)	927,496	
7	5 OR 6	968,228	
8	3 AND 4 AND 7	902	
9	8 NOT (ti(Coronary) OR ab(Coronary))	627	
10	8 AND Date Publication: 2019-12-01 - 2020-12-31	461	

Web of Science 2020-12-04

Database: Web of Science, Clarivate Analytics

Date: 2020-12-04

Number	Search terms	Results	Comment
1	TS=(Adolesc* OR Preadolesc* OR Pre-adolesc* OR Boy OR Boys OR Boyhood OR Girl* OR Child OR Children OR Childhood OR Kid OR Kids OR Toddler* OR Preschool* OR Pre-school* OR Teen* OR Young* OR Youth* OR School* OR "Junior high*" OR "High-school*" OR Highschool* OR "Senior high" OR Schoolchild* OR Kindergarten* OR Playgroup* OR Play-group* OR Playschool*)	3,398,609	
2	TS=(Corona* OR cov2 OR COVID19 OR COVID-19 OR nCov OR Pandemic* OR SARS-CoV-2 OR 2019-nCoV*)	752,219	
3	TS=("Mental Disorder*" OR "Mental Disease*" OR "Mental Illness*" OR "Mental Instabilit*" OR "Psychiatric Disorder*" OR "Psychiatric Disease*" OR "Psychiatric Illness*" OR "Psychiatric Diagnosis" OR "Mood disorder*" OR "Affective Disorder*" OR "Behavior Disorder*" OR Depressi* OR "Mental Health" OR "Well Being" OR "Well-Being" OR Anxiet* OR Nervous* OR Worr* OR Stress* OR Distress* OR Anger* OR Angry OR Suicide*)	3,508,561	
4	#1 AND #2 AND #3	5,772	
5	#4 NOT TS=(Coronary)	1,660	
6	#5 AND Timespan=2019-2020	1,054	
